# MC1R signaling through the cAMP-CREB/ATF-1 and ERK-NFκB pathways accelerates G1/S transition promoting breast cancer progression

**DOI:** 10.1038/s41698-023-00437-1

**Published:** 2023-09-07

**Authors:** Vipin Shankar Chelakkot, Kiara Thomas, Todd Romigh, Andrew Fong, Lin Li, Shira Ronen, Shuyang Chen, Pauline Funchain, Ying Ni, Joshua Arbesman

**Affiliations:** 1 Department of Cancer Biology, Lerner Research Institute, Cleveland Clinic, Cleveland, OH USA; 2 Center for Immunotherapy & Precision Immuno-Oncology, Lerner Research Institute, Cleveland Clinic, Cleveland, OH USA; 3 Department of Anatomic Pathology, Pathology and Laboratory Medicine Institute, Cleveland Clinic, Cleveland, OH USA; 4 Department of Hematology & Oncology, Taussig Cancer Center, Cleveland Clinic, Cleveland, OH USA; 5 Department of Dermatology, Dermatology and Plastic Surgery Institute, Cleveland Clinic, Cleveland, OH USA; 6 Department of Dermatology, Cleveland Clinic Lerner College of Medicine, Case Western Reserve University, Cleveland, OH USA

**Keywords:** Oncogenes, Breast cancer

## Abstract

MC1R, a G-protein coupled receptor, triggers ultraviolet light-induced melanin synthesis and DNA repair in melanocytes and is implicated in the pathogenesis of melanoma. Although widely expressed in different tissue types, its function in non-cutaneous tissue is relatively unknown. Herein, we demonstrate that disruptive *MC1R* variants associated with melanomagenesis are less frequently found in patients with several cancers. Further exploration revealed that breast cancer tissue shows a significantly higher *MC1R* expression than normal breast tissue, and knocking down MC1R significantly reduced cell proliferation in vitro and in vivo. Mechanistically, MC1R signaling through the MC1R-cAMP-CREB/ATF-1 and MC1R-ERK-NFκB axes accelerated the G1-S transition in breast cancer cells. Our results revealed a new association between MC1R and breast cancer, which could be potentially targeted therapeutically. Moreover, our results suggest that MC1R-enhancing/activating therapies should be used cautiously, as they might be pro-tumorigenic in certain contexts.

## Introduction

Melanocortin 1 receptor (MC1R) is a G-protein-coupled receptor (GPCR) that regulates skin pigmentation, cell proliferation, and apoptosis^1^. The binding of α-melanocyte-stimulating hormone (αMSH) to MC1R stimulates cAMP synthesis^2^, inducing the production of dark, photo-protective eumelanin that colors the skin, hair, and iris. Disruptive germline *MC1R* variants yielding poorly photo-protective pheomelanin^3,4^ are associated with red hair and sun sensitivity and are major genetic determinants of cutaneous melanoma risk^5,6^. Active MC1R signaling also triggers the repair of UV-induced DNA damage^7^ and slows down melanocyte proliferation^8^, rendering a protective effect on melanomagenesis. Contrastingly, it has also been reported that αMSH shows a weak mitogenic effect in melanocytes in culture^9,10^, albeit only a transient increase in DNA synthesis without a significant increase in cell number was observed when the melanocytes were stimulated with αMSH alone^11–13^. Interestingly, inherited *MC1R* variants are associated with survival benefits in melanoma patients^14–17^.

Although MC1R expression has been reported in several cells other than melanocytes^18–20^, its functions in those cells remain largely unknown. MC1R is reportedly involved in the melanocortin system, modulating several physiological and immunomodulatory functions^21^. A recent study reported an evolutionarily conserved role of MC1R in the development of muscles, cartilage, and other internal organs^22^. However, few studies have focused on the expression of MC1R and its role in the development and progression of non-cutaneous cancers. In this study, we sought to determine the role of MC1R in the pathogenesis of non-cutaneous cancers. Preliminary analysis of The Cancer Genome Atlas (TCGA) data suggested that active MC1R signaling might contribute to the pathogenesis of several cancers. Further exploration revealed that MC1R signaling accelerates breast cancer cell proliferation and contributes to tumorigenicity, identifying it as a therapeutic target for breast and, potentially, other cancers.

## Results

### MC1R is widely expressed in several tissues and cancers

*MC1R* gene expression analysis in human tissues using the genotype-tissue expression (GTEx) portal revealed expression in almost all tissues (Supplementary Fig. 1a). Similarly, analysis of the Human Protein Atlas database^23^ (Supplementary Fig. 1b) and the cancer cell line encyclopedia using cBioPortal^24,25^ (Supplementary Fig. 1c) revealed high *MC1R* expression in several non-melanoma cancer cell lines, suggesting that MC1R might be significant in the development and progression of cancers other than melanoma.

### Disruptive germline *MC1R* variants are less frequently found in patients with non-melanoma cancers

**Fig. 1:**
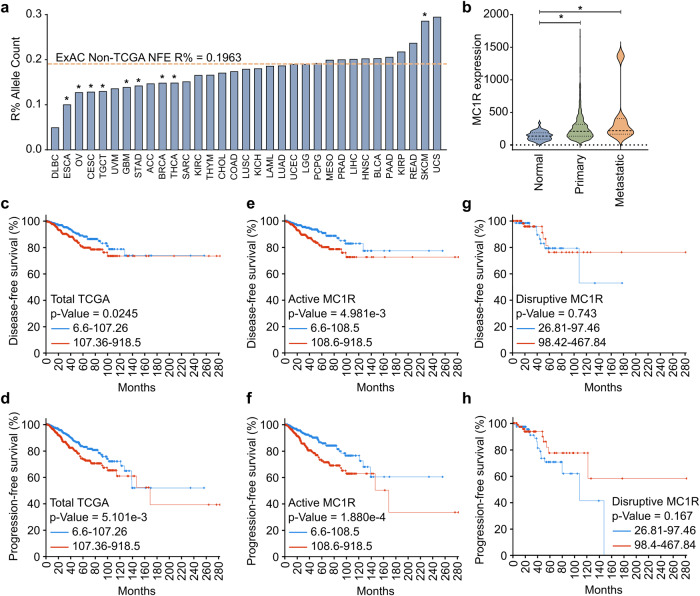
*MC1R* expression is associated with breast cancer. **a** The *MC1R* R% allele frequency across cancers in The Cancer Genome Atlas (TCGA). The orange dashed line indicates the *R*% allele frequency in control ExAC non-TCGA non-finnish European (NFE) Ancestry population. *p < 0.05 (Fisher’s exact test with Benjamini–Hochberg (BH) multiple comparisons adjustment) compared to the control population. The cancer type abbreviations are expanded and listed in Supplementary Table 2. **b** *MC1R* mRNA expression in normal breast, primary, and metastatic breast cancers. Data obtained from TCGA. The dashed lines in the violin plot show the median and the 25^th^ and 75^th^ percentiles. *p < 0.05 (one-way ANOVA with Dunnett’s multiple comparisons). **c**–**h** Disease-free survival (DFS) (**c**, **e**, **g**) and progression-free survival (PFS) (**d**, **f**, **h**) of all patients with breast cancer in TCGA (DFS: low expression *n* = 462, high expression *n* = 477; PFS: low expression *n* = 539, high expression *n* = 541) (**c**, **d**), patients carrying active *MC1R* variants (DFS: low expression *n* = 395, high expression *n* = 408; PFS, low expression *n* = 461, high expression *n* = 462) (**e**, **f**), and patients carrying a disruptive MC1R variant (DFS, low expression *n* = 67, high expression *n* = 69; PFS, low expression *n* = 78, high expression *n* = 79) (**g**, **h**) based on MC1R expression. *p*-Value calculated using the Log-rank test.

To study the effect of the disruptive *MC1R* variants on different cancer types, we extracted *MC1R* DNA germline variants from the whole-exome sequences of 10,391 patients with cancer housed in TCGA. Our inclusion criteria for disruptive germline *MC1R* alleles were defined according to those outlined previously^5^ (listed in Supplementary Table. 1), with inclusion limited to white patients as disruptive germline *MC1R* alleles are significantly more prevalent in the Caucasian population. The R% allele frequency (percent of total *MC1R* alleles displaying disruptive (R) allele variants) was determined for each cancer type and our control population. Fisher’s exact analyses on each cancer type showed that nine of the 33 cancer types displayed significantly different R% counts compared to the “general population,”—the exome aggregation consortium (ExAC) non-TCGA non-finnish European ancestry population (ExAC Non-TCGA NFE; R% allele frequency = 0.1963) (Fig. 1a, Supplementary Table 2). A significantly higher incidence of *R*% count by allele was observed in skin cutaneous melanoma, as reported previously (odd’s ratio (OR) = 1.6418)^5,6^. Of the cancers displaying significantly different *R*% allele frequencies, surprisingly, eight cancers (thyroid carcinoma (OR = 0.7177), breast invasive carcinoma (OR = 0.7161), stomach adenocarcinoma (OR = 0.6805), glioblastoma multiforme (OR = 0.6631), testicular germ cell tumors (OR = 0.6128), cervical squamous cell carcinoma and endocervical adenocarcinoma (OR = 0.6070), ovarian serous cystadenocarcinoma (OR = 0.6001), and esophageal carcinoma (OR = 0.4591)) exhibited significantly decreased *R*% allele frequencies (Fig. 1a, Supplementary Table 2). This demonstrated that the melanoma-causing, disruptive *MC1R* germline variants are less frequently seen in individuals with these cancers, implying that, unlike in melanoma, these disruptive germline *MC1R* variants might render a protective effect on the development of these cancers. Conversely, the active/wild-type *MC1R* is more frequently observed in patients with these cancers, suggesting that active MC1R signaling might contribute to the development of non-melanoma cancers.

### Active MC1R is associated with breast cancer progression

To further characterize this new association, we selected breast cancer, which displayed one of the highest statistically significant decreases in the *R*% allele frequency (Supplementary Table 2). An analysis of the Breast Cancer Genome Guided Therapy Study (BEAUTY) dataset of patients with breast cancer revealed an *R*% allele frequency of 0.1591, which was less than the *R*% allele frequency of the control population (0.1963), although not statistically significant, likely due to the smaller sample size than that used in TCGA (Supplementary Table 3).

An analysis of *MC1R* expression in different breast cancer tissue types in TCGA revealed a significantly higher expression in breast cancer tissue compared to normal breast tissue (Fig. 1b). Additionally, *MC1R* expression trended to be further elevated in metastatic breast cancer (Fig. 1b). Furthermore, patients with lower *MC1R* expression levels showed better disease-free survival (DFS) (Fig. 1c) and progression-free survival (PFS) (Fig. 1d). At the 5-year mark, patients with higher *MC1R* expression had worse outcomes and showed an absolute difference of 10% in the DFS and PFS rates compared to the patients with lower *MC1R* expression (Fig. 1c, d). On segregating the patients based on their *MC1R* germline variants, higher expression levels showed a significantly lower DFS (Fig. 1e) and PFS (Fig. 1f) among the patients expressing the active *MC1R* germline variant. At the 5-year mark, patients with higher *MC1R* expression had a worse outcome and showed an absolute difference of 12% in the DFS and PFS rates compared to those with lower *MC1R* expression among the patients with active *MC1R* germline variant (Fig. 1e, f). No significant associations were observed between the *MC1R* high and low groups among the patients with the disruptive *MC1R* germline variants (Fig. 1g, h).

Since the frequency of *MC1R* variants is highly associated with ethnicity^26^, we further analyzed the population-specific differences in breast cancer survival and the presence of *MC1R* disruptive variants (Supplementary Fig. 2). The Black or African American population showed a significantly higher expression of *MC1R* compared to the rest (Supplementary Fig. 2a), and 85.4% of the population with disruptive germline *MC1R* variants were white individuals (Supplementary Fig. 2b). A significantly higher *MC1R* expression was observed in primary breast cancer (and metastatic breast cancers among the white population) compared to normal breast tissue in both the Black or African American and white populations (Supplementary Fig. 2c). The other ethnic groups were not analyzed due to the lack of data. Additionally, low *MC1R* expression showed a significantly better PFS and DFS among white individuals carrying the active MC1R variant, while no significant association with survival was observed in the other ethnic groups assessed (Supplementary Fig. 2d–g).

Additionally, since the *MC1R* gene is located on chromosome 16 (16q24.3), which is often altered in breast cancer, we compared *MC1R* expression with 16q and putative copy-number alterations in the breast cancer population and identified a significant association between *MC1R* expression and copy number variation (Supplementary Fig. 2h).

### MC1R expression accelerates breast cancer progression in vitro, in vivo, and in human breast cancer samples

**Fig. 2:**
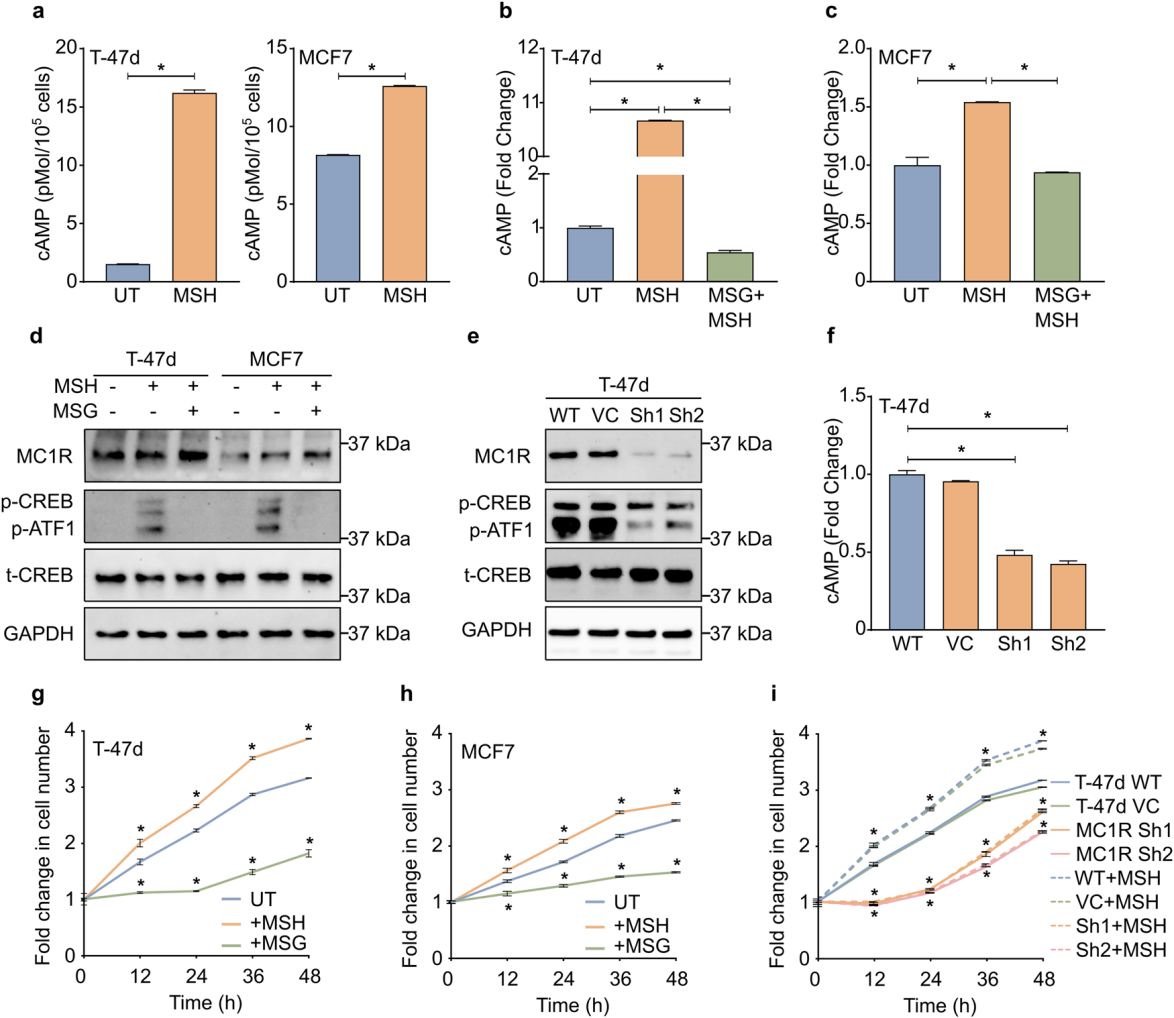
MC1R signaling is active in breast cancer cells and promotes breast cancer cell progression in vitro. **a** Mean ± SEM cAMP levels in T-47d and MCF7 cells treated with or without 0.2 μM NDP-MSH from three independent experiments. *p < 0.05 (unpaired Student’s *t*-test). **b**, **c** Mean ± SEM percentage fold change in cAMP levels in (**b**) T-47d and (**c**) MCF7 cells treated with or without 20 μM MSG-606 and stimulated with 0.2 μM NDP-MSH compared to those left untreated (UT) from three independent experiments. *p < 0.05 (one-way ANOVA with Tukey’s multiple comparisons). **d** T-47d and MCF7 cells were treated with 0.2 μM NDP-MSH or pretreated with 20 μM MSG-606, followed by stimulation with 0.2 μM NDP-MSH or left untreated. Representative western blot showing MC1R expression and downstream signaling in T-47d, MCF7 cells. GAPDH is shown as the loading control. Western blot quantification plots are shown in Supplementary Fig. 3a. **e** WT T-47d (WT) and MC1R knockdown (KD) T-47d (MC1R Sh1 and MC1R Sh2) cell lines were treated with 0.2 μM NDP-MSH. Representative western blot showing MC1R expression and downstream signaling. GAPDH is shown as the loading control. Western blot quantification plots are shown in Supplementary Fig. 3b. **f** Mean ± SEM fold change in cAMP levels in MC1R-KD T-47D cells treated with 0.2 μM NDP-MSH from 3 independent experiments. *p < 0.05 (one-way ANOVA with Tukey’s multiple comparisons). **g**, **h** Mean ± SEM fold change in cell number in (**g**) T-47d and (**h**) MCF7 cells treated either with 0.2 μM NDP-MSH or 20 μM MSG-606 or left untreated (UT) over time from three independent experiments. *p < 0.05 (two-way ANOVA with Dunnett’s multiple comparisons) compared to UT at the indicated time points. **i** Mean ± SEM fold change in cell number of WT T-47d cells and MC1R-KD T-47d cells (MC1R Sh1 and MC1R Sh2) treated with or without 0.2 μM NDP-MSH. (VC, vector control) over time from three independent experiments. *p < 0.05 (two-way ANOVA with Dunnett’s multiple comparisons) compared to T-47d WT at the indicated time points.

To understand the impact of MC1R on breast cancer, we used T-47d and MCF7, two breast cancer cell lines that showed high *MC1R* expression (Supplementary Fig. 1b). An analysis of breast cancer cell lines using DepMap and cBioPortal showed that T-47d and MCF7 did not carry any *MC1R* mutations. In melanocytes, αMSH binding to MC1R triggers Gsα-dependent stimulation of adenylyl cyclase, leading to increased cellular cAMP levels, resulting in the activation of PKA and PKA-dependent phosphorylation of CREB^27,28^. We sought to determine whether this signaling pathway is active in breast cancer cells. To determine whether MC1R signaling was active in the breast cancer cell lines, T-47d and MCF7, we treated the cells with the MC1R agonist and α-MSH analog, Nle4-D-Phe7-α-MSH (NDP-MSH), or the MC1R antagonist, MSG-606^29–31^, and evaluated the changes in intracellular cAMP levels. Treatment with MSH significantly increased cAMP levels in both T-47d and MCF7 cells, while pretreatment with MSG-606 significantly attenuated the NDP-MSH-stimulated increase in cAMP (Fig. 2a–c). Further, western blot analysis confirmed that MC1R is expressed in T-47d and MCF7 cells (Fig. 2d, Supplementary Fig. 3a) and that stimulation with NDP-MSH significantly increased CREB phosphorylation (Fig. 2d, Supplementary Fig. 3a). To further confirm MC1R activity in breast cancer cell lines, we generated MC1R-knock down (MC1R-KD) T-47d cells (Fig. 2e, Supplementary Fig. 3b) and evaluated intracellular cAMP levels and MC1R downstream signaling. MC1R downregulation significantly reduced NDP-MSH-stimulated cAMP generation (Fig. 2f) and CREB phosphorylation in T-47d cells (Fig. 2e, Supplementary Fig. 3b). Subsequently, we determined the effect of treatment with NDP-MSH and MSG-606 on breast cancer cell growth. Treatment with NDP-MSH increased cell proliferation, while MC1R inhibition using MSG-606 decreased cell proliferation in T-47d and MCF7 cells (Fig. 2g, h). Further, the MC1R-KD T-47d cells showed decreased proliferation and did not respond to NDP-MSH stimulation, while wild-type (WT) T-47d showed accelerated growth with NDP-MSH stimulation (Fig. 2i).

**Fig. 3:**
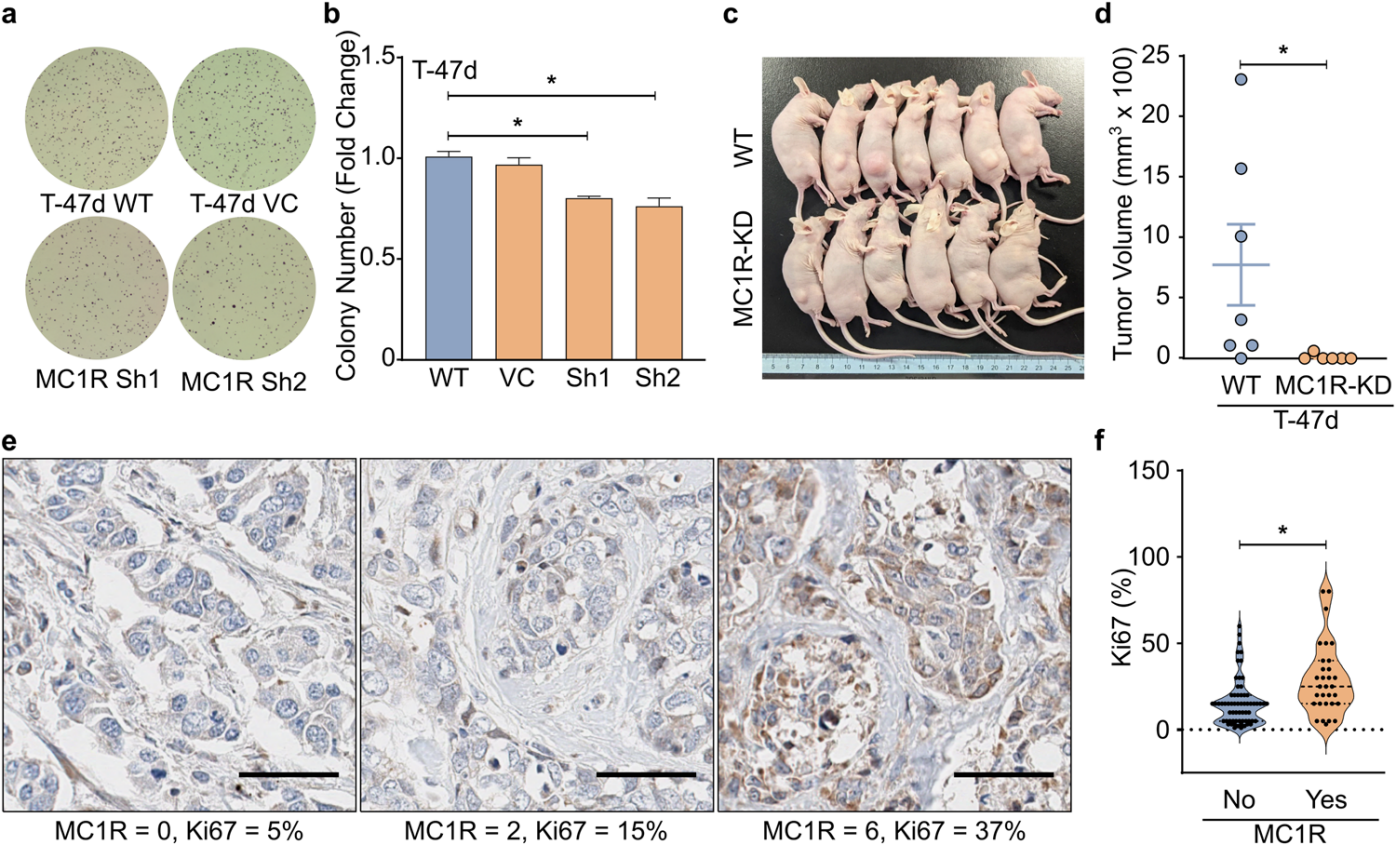
MC1R promotes tumorigenicity and breast cancer progression in vitro and in vivo. **a**, **b** Soft agar colony formation for WT T-47d and MC1R-KD T-47d (MC1R Sh1 and MC1R Sh2) cells. **a** Representative images of the soft agar wells. **b** Mean ± SEM fold change in the number of soft agar colonies from three independent experiments. *p < 0.05 (one-way ANOVA with Dunnett’s multiple comparisons). **c**, **d** Athymic nude mice carrying 17β-estradiol pellets were subcutaneously injected with WT T-47d or MC1R-KD T-47d cells, and tumor development was monitored. **c** Mice pictured on day 24 post-implant. **d** Tumor volume on day 24 post-implant in mice that received the WT T-47d (*n* = 7) or MC1R-KD T-47d (*n* = 6) cells are shown; the error bar shows mean ± SEM. *p < 0.05 (unpaired Student’s *t*-test). **e** Representative micrograph (40×) showing breast cancer tissue samples with different MC1R staining scores (0, 2, 6) and Ki67 expression in the breast cancer tissue microarray. Scale bar = 50 μm. **f** Plot comparing MC1R expression and Ki67 expression. The dashed lines in the violin plot show the median and the 25th and 75th percentiles. *p < 0.05 unpaired Student’s *t*-test.

To evaluate the effect of MC1R signaling on tumorigenicity, we performed a soft agar colony formation assay using the WT T-47d and MC1R-KD T-47d cells. The MC1R-KD T-47d lines formed significantly fewer colonies on soft agar compared to the WT cells (Fig. 3a, b), suggesting lower tumorigenicity. To confirm the tumorigenic effect of MC1R, we xenografted WT or MC1R-KD T-47d cells subcutaneously into the hind flanks of athymic nude mice carrying a subcutaneous 17β-estradiol pellet on the back of their necks to support tumor formation. Six of the seven mice that received WT T-47d cells formed palpable tumors by day 10, while none of the mice that received the MC1R-KD T-47d cells formed tumors. One mouse that received the MC1R-KD T-47d cells died of an unrelated cause on day 15 post-implantation, with no tumors at death. On day 24, six of the seven (85.7%) mice that received the WT T-47d cells had tumors, while only one of the mice that received the MC1R-KD T-47d cells had a tumor (Fig. 3c, d), suggesting that MC1R signaling was critical for imparting tumorigenicity in T-47d cells.

To better understand the effect of MC1R in breast cancer progression, we evaluated MC1R expression in a human breast cancer tumor microarray (TMA). The TMA included breast cancers of different pathological and clinical grades and cancer-adjacent tissue. Differential MC1R protein expression was observed across the TMA, indicating that MC1R is expressed in many breast cancers (Fig. 3e). Tumors that expressed MC1R showed significantly higher expression of Ki67, a proliferation marker (Fig. 3f), suggesting that MC1R might be associated with breast cancer cell proliferation. Additionally, we noted elevated MC1R expression in HER2-negative cancer samples (Supplementary Fig. 3c). In line with this, an analysis of *MC1R* mRNA expression across different breast cancer types in TCGA revealed a significantly lower *MC1R* expression in HER2-positive breast cancers (Supplementary Fig. 3d).

### MC1R downregulation decelerates G1-S transition

**Fig. 4:**
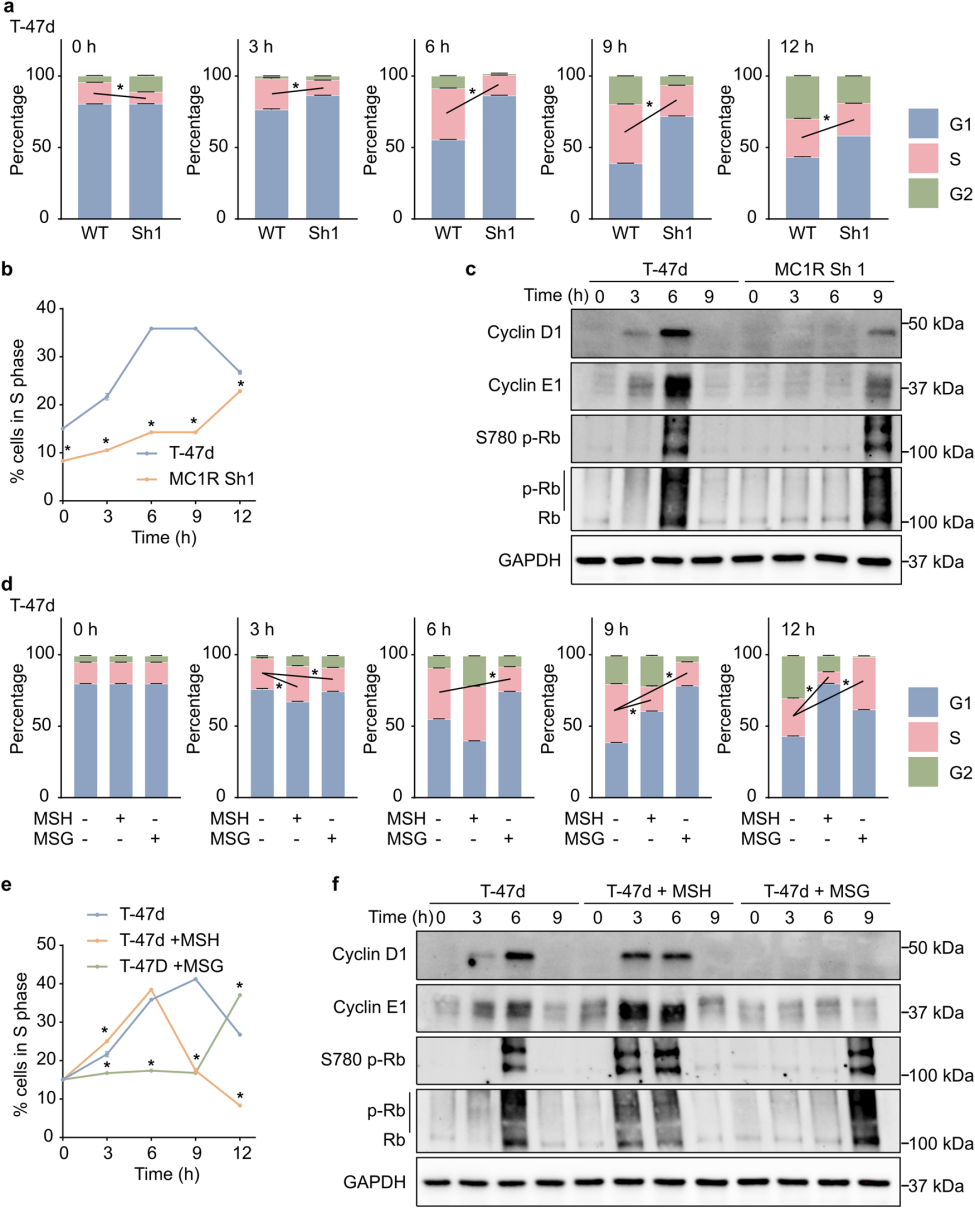
MC1R downregulation delays G1–S progression in breast cancer cells. **a**–**c** Wild-type (WT) T-47d and MC1R-Knockdown (KD) T-47d cells were synchronized to the G1 phase by a double-thymidine block and then released. **a** Mean ± SEM percentage of cells in the G1, S, and G2 phases at 0, 3, 6, 9, and 12 h post-release. *p < 0.05 (unpaired Student’s *t*-test). **b** Mean ± SEM percentage of cells in the S phase across time. *p < 0.05 (one-way ANOVA with Dunnett’s multiple comparisons). **c** Representative western blot showing Cyclin D1, Cyclin E1, Ser780 p-RB, Rb, and GAPDH (loading control). Western blot quantification plots are shown in Supplementary Fig. 4a. **d**–**f** WT T-47d cells were treated with 0.2 μM NDP-MSH or 20 μM MSG-606 or left untreated after releasing from a double-thymidine block. **d** Mean ± SEM percentage of cells in the G1, S, and G2 phases at 0, 3, 6, 9, and 12 h post-release. *p < 0.05 (unpaired Student’s *t*-test). **e** Mean ± SEM percentage of cells in the S phase across time. *p < 0.05 (one-way ANOVA with Dunnett’s multiple comparisons). **f** Representative western blot showing Cyclin D1, Cyclin E1, Ser780 p-RB, Rb, and GAPDH (loading control). Western blot quantification plots are shown in Supplementary Fig. 4b.

We performed a cell cycle progression analysis to further characterize the lower cell proliferation rate in the MC1R-KD T-47d cells. Before the experiment, all cells were synchronized to the G1 phase by a double-thymidine block. The MC1R-KD T-47d cells took significantly longer to transition from the G1 to the S phase of the cell cycle post-release from the double-thymidine block (Fig. 4a, b). In line with this, the WT T-47d cells started expressing Cyclin D1 and Cyclin E1 3 h post-release, which was further upregulated at 6 h post-release (Fig. 4c, Supplementary Fig. 4a). A significant increase in Rb hyper-phosphorylation was also observed 6 h post-release (Fig. 4c, Supplementary Fig. 4a). These suggested that the cells progressed to the S phase at around 3–6 h post-release. On the other hand, the MC1R-KD T-47d cells started showing Cyclin D1 and Cyclin E1 expression and Rb hyper-phosphorylation only at 9 h post-release (Fig. 4c, Supplementary Fig. 4a), confirming a delayed transition to S phase. Additionally, the WT T-47d cells showed accelerated G1-S transition with NDP-MSH stimulation, while a single treatment with MSG-606 prolonged the time taken to transition from the G1 to the S phase (Fig. 4d, e). Accordingly, NDP-MSH-stimulated WT T-47d cells showed significantly increased Cyclin D1 and Cyclin E1 expression and Rb hyper-phosphorylation 3 h post-release from the double-thymidine block, compared to the non-stimulated cells (Fig. 4f, Supplementary Fig. 4b), while MSG-606-treated cells did not express Cyclin D1 even at 9 h post-release (Fig. 4f, Supplementary Fig. 4b), suggesting a delay in G1 to S transition. However, Cyclin E1 expression and Rb hyper-phosphorylation were observed 9 h post-release (Fig. 4f, Supplementary Fig. 4b).

### MC1R regulates cell proliferation through the MC1R-CREB-Cyclin D1 and MC1R-ERK-NFκB axes

**Fig. 5:**
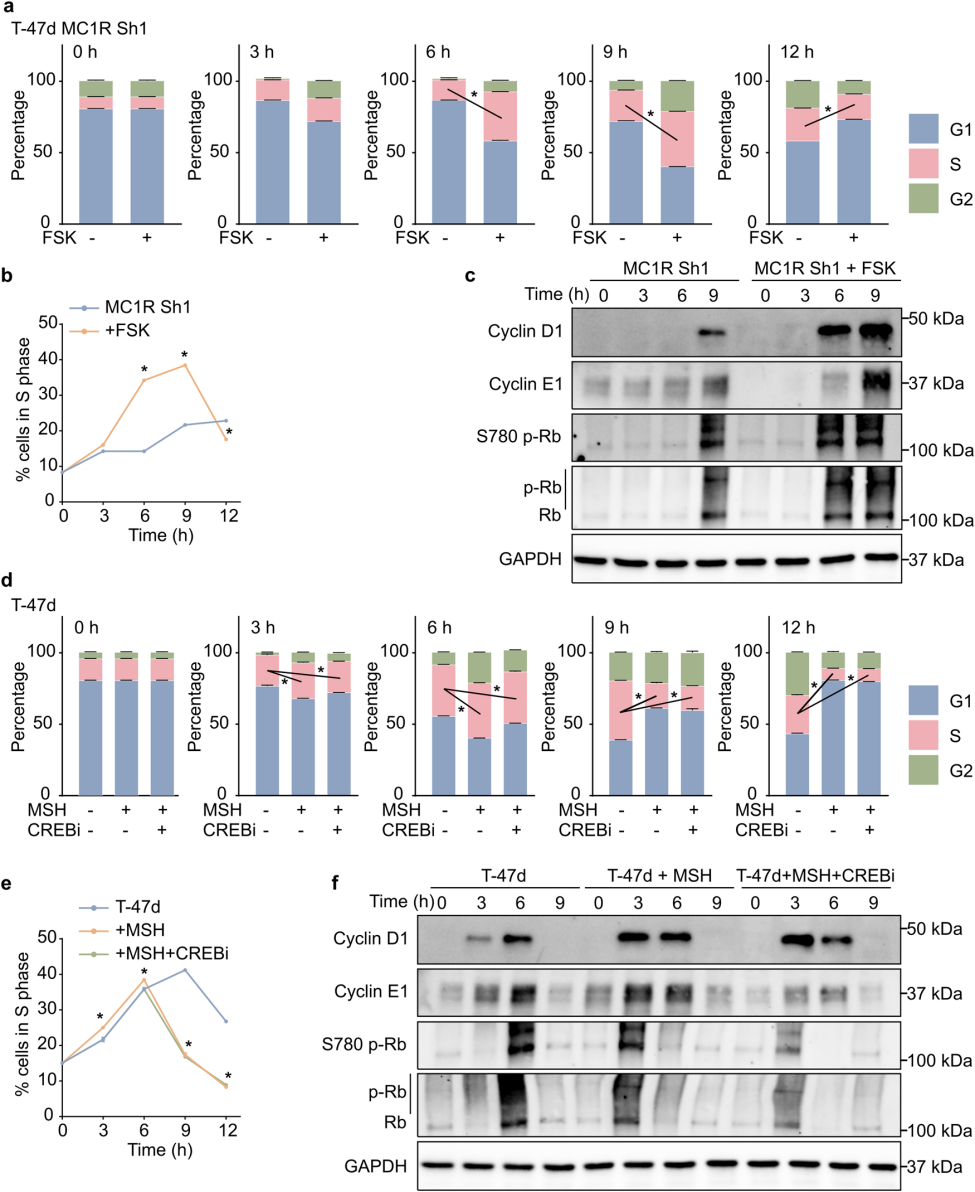
MC1R signaling through MC1R-cAMP-CREB contributes to the accelerated G1-S transition in breast cancer cells. **a–c**. MC1R-KD T-47d cells were synchronized to the G1 phase by a double-thymidine block and then released with or without treatment with 25 μM FSK. (**a**) Mean ± SEM percentage of cells in the G1, S, and G2 phases at 0, 3, 6, 9, and 12 h post-release. *p < 0.05 (unpaired Student’s *t*-test). (**b**) Mean ± SEM percentage of cells in the S phase across time. *p < 0.05 (one-way ANOVA with Dunnett’s multiple comparisons). (**c**) Representative western blot showing Cyclin D1, Cyclin E1, Ser780 p-Rb, Rb, and GAPDH (loading control). Western blot quantification plots are shown in Supplementary Fig. 4d. **d–f**. WT T-47d cells were treated with 0.2 μM NDP-MSH or 0.2 μM NDP-MSH + 5 μM 666-15 (CREBi) or left untreated after releasing from a double-thymidine block. (**d**) Mean ± SEM percentage of cells in the G1, S, and G2 phases at 0, 3, 6, 9, and 12 h post-release. *p < 0.05 (unpaired Student’s *t*-test). (**e**) Mean ± SEM percentage of cells in the S phase across time. *p < 0.05 (one-way ANOVA with Dunnett’s multiple comparisons). (**f**) Representative western blot showing Cyclin D1, Cyclin E1, Ser780 p-Rb, Rb, and GAPDH (loading control). Western blot quantification plots are shown in Supplementary Fig. 4e.

To evaluate whether the delay in G1–S transition was due to reduced cAMP generation in the MC1R-KD T-47D cells, we treated them with forskolin (FSK), which activates adenylyl cyclase and increases intracellular cAMP. FSK treatment significantly increased intracellular cAMP levels (Supplementary Fig. 4c) and accelerated cell cycle progression into the S phase in MC1R-KD T-47d cells (Fig. 5a, b). Accordingly, significantly higher Cyclin D1 and Cyclin E1 levels were observed at 6 and 9 h post-release from the double-thymidine block in FSK-treated MC1R-KD T-47d cells, while the untreated cells showed very little Cyclin D1 and Cyclin E1 expression only at 9 h post-release (Fig. 5c, Supplementary Fig. 4d). Similarly, FSK-treated MC1R-KD-T-47d cells showed elevated Rb hyper-phosphorylation 6 and 9 h post-release, while the untreated cells showed Rb hyper-phosphorylation only at 9 h post-release (Fig. 5c, Supplementary Fig. 4d). These suggested that active MC1R signaling-induced cAMP generation might trigger CREB activation and accelerate the G1 to S transition. However, the accelerated G1 to S transition of WT T-47d cells after NDP-MSH stimulation could only be partially attenuated with CREB inhibition with the CREB inhibitor, 666-15 (Fig. 5d, e). In line with this, no significant changes in Cyclin D1 expression at 3 h post-release were observed between T-47d cells stimulated with NDP-MSH and those treated with NDP-MSH and 666-15 (Fig. 5f, Supplementary Fig. 4e). However, a significant decrease in Cyclin D1 expression at 6 h post-release was observed in the cells treated with NDP-MSH and 666-15 compared to those treated with NDP-MSH alone (Fig. 5f, Supplementary Fig. 4e). Treatment with 666-15 decreased Cyclin E1 expression at 3 h and 6 h post-release compared to the control T-47d cells and those stimulated with NDP-MSH. Although the cells treated with 666-15 showed a significant increase in Rb hyper-phosphorylation at 3 h post-release compared to the untreated control cells, it was significantly attenuated compared to the NDP-MSH-stimulated cells (Fig. 5f, Supplementary Fig. 4e). These results showed that compared to the NDP-MSH-stimulated cells, fewer cells treated with 666-15 and stimulated with NDP-MSH were transitioning to the S phase at around 3 h post-release. These suggested that MC1R-signaling-triggered accelerated cell cycle progression was only partially mediated through the MC1R-cAMP-CREB signaling axis, and other mechanisms were also at play.

**Fig. 6:**
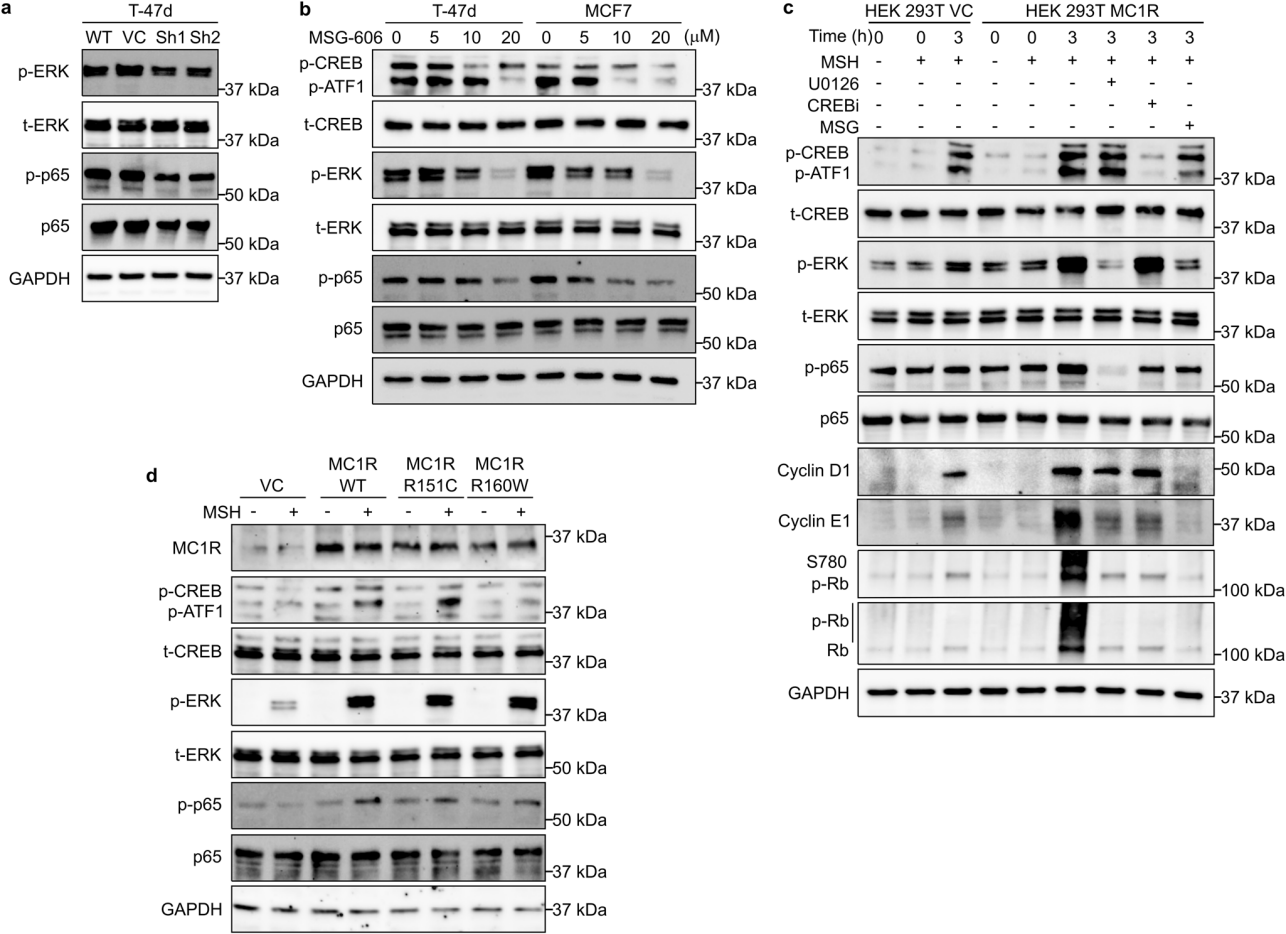
MC1R signaling through MC1R-cAMP-CREB/ATF and MC1R-ERK-NFκB axes promote G1/S transition. **a** WT T-47d (WT) and MC1R knockdown (KD) T-47d (MC1R Sh1 and MC1R Sh2) cell lines were stimulated with 0.2 μM NDP-MSH. Representative western blot showing p-ERK, t-ERK, p-p65 NFκB, and p65 NFκB, and GAPDH (loading control) in T-47d WT (wild-type), VC (vector control), and MC1R-KD T-47d (Sh1 and Sh2) cells. Western blot quantification plots are shown in Supplementary Fig. 5a. **b** T-47d and MCF7 cells were treated with different concentrations of MSG-606, as indicated, before stimulation with 0.2 μM NDP-MSH. Representative western blot showing p-CREB, p-ATF-1, t-CREB, p-ERK, t-ERK, p-p65 NFκB, p65 NFκB, and GAPDH (loading control). Western blot quantification plots are shown in Supplementary Fig. 5b. **c** HEK 293 T VC (vector control) and HEK 293 T cells transiently overexpressing MC1R (HEK 293 T MC1R) were serum-starved overnight and then released with 0.2 μM NDP-MSH with or without treatment with a MEK inhibitor, 5 μM U0126, a CREB inhibitor, 5 μM 666-15, or 20 μM MSG-606. Representative western blot showing p-CREB, p-ATF-1, t-CREB, p-ERK, t-ERK, p-p65 NFκB, and p65 NFκB, Cyclin D1, Cyclin E1, Ser780 p-Rb, Rb, and GAPDH (loading control). Western blot quantification plots are shown in Supplementary Fig. 6c. **d** HEK 293 T VC (vector control) and HEK 293 T cells transiently overexpressing wild-type MC1R (MC1R WT) or the MC1R variants (MC1R R151C and MC1R R160W) were serum-starved overnight and then released with or without 0.2 μM NDP-MSH. Representative western blot showing p-CREB, p-ATF-1, t-CREB, p-ERK, t-ERK, p-p65 NFκB, p65 NFκB, and GAPDH (loading control). Western blot quantification plots are shown in Supplementary Fig. 6d.

Previous studies have reported a cross-talk between MC1R and the ERK signaling pathways^32^. Therefore, we examined whether active MC1R signaling is transduced through ERK in breast cancer cells. We also evaluated p65 NFκB expression and phosphorylation as they are reported to be regulated by ERK^33^ and are implicated in Cyclin D1 expression in breast cancer^34^. Western blot analysis showed that MC1R downregulation decreased CREB and ATF-1 phosphorylation (Fig. 2e) in the WT T-47d cells, confirming active signaling through the MC1R-CREB axis. Further, MC1R downregulation also significantly decreased ERK and p-65 NFκB phosphorylation by about 40% (Fig. 6a, Supplementary Fig. 5a). Additionally, treatment with an MC1R antagonist, MSG-606, significantly decreased the phosphorylation of ERK and p-65 NFκB in addition to CREB and ATF-1 in a dose-dependent manner in T-47d and MCF7 cells (Fig. 6b, Supplementary Fig. 5b). Further, ERK inhibition using U0126 in NDP-MSH-stimulated cells significantly reduced Cyclin D1 and Cyclin E1 expression and Rb hyper-phosphorylation at 3 h and 6 h post-release compared to NDP-MSH-stimulated cells and showed a delayed G1/S progression compared to the NDP-MSH-stimulated cells (Supplementary Fig. 5c–f). These suggested that active MC1R signaling through two signaling pathways, the MC1R-cAMP-CREB/ATF-1 and the MC1R-ERK-NFκB pathways, controlled growth and proliferation in breast cancer cells. To determine whether MC1R-mediated ERK phosphorylation was mediated through the MSH-stimulated increase in cAMP, we treated WT T-47d and MC1R-KD-T-47d cells with FSK. Predictably, FSK treatment significantly increased CREB phosphorylation in both WT and MC1R-KD-T-47d cells (Supplementary Fig. 5g, h). However, the FSK-induced increase in cAMP did not significantly affect ERK or p65 NFκB phosphorylation (Supplementary Fig. 5g, h), suggesting ERK pathway activation downstream of MC1R is most likely cAMP-independent.

To eliminate the effects of other oncogenic pathways in breast cancer cells and to confirm the mechanisms contributing to MC1R-driven cell proliferation, we used HEK 293 T cells transiently overexpressing MC1R (Supplementary Fig. 6a, b). NDP-MSH-stimulation significantly increased CREB and ERK phosphorylation (Fig. 6c Supplementary Fig. 6c) in HEK 293 T cells 3 h post-stimulation; no significant change in p65 NFκB phosphorylation was observed. The NDP-MSH-stimulated cells also showed significant increases in Cyclin D1 and Cyclin E1 levels and Ser780 Rb phosphorylation. However, no significant increase in Rb hyper-phosphorylation was observed (Fig. 6c, Supplementary Fig. 6b). These suggested that these cells were beginning to transition to the S phase 3 h post-NDP-MSH stimulation. MC1R-overexpressing HEK 293 T cells showed significantly higher phosphorylation of ERK, CREB, and p65 NFκB 3 h post-NDP-MSH stimulation compared to the HEK 293 T cells (Fig. 6c, Supplementary Fig. 6b). Moreover, the MC1R-overexpressing HEK 293 T cells showed significant upregulation of Cyclin D1 and Cyclin E1 expression and Rb hyper-phosphorylation 3 h post-NDP-MSH stimulation compared to the controls (Fig. 6c, Supplementary Fig. 6b), suggesting an accelerated transition to S phase. Treatment with CREB inhibitor, 666-15, or MEK inhibitor, U0126, only affected the respective signaling axes in NDP-MSH-stimulated MC1R-overexpressing HEK 293 T cells and significantly affected NDP-MSH-stimulation-induced Cyclin D1 and Cyclin E1 expression and Rb hyper-phosphorylation (Fig. 6c, Supplementary Fig. 6b). On the other hand, MSG-606 (MC1R inhibitor) decreased phosphorylation of CREB, ATF-1, ERK, and p65 NFκB in NDP-MSH-treated MC1R-overexpressing HEK 293 T cells (Fig. 6c, Supplementary Fig. 6b). Additionally, complete attenuation of the NDP-MSH-stimulated increase in Cyclin D1 and Cyclin E1 levels and Rb hyper-phosphorylation was observed (Fig. 6c, Supplementary Fig. 6b). These results show that MC1R signaling through the MC1R-cAMP-CREB/ATF-1 and MC1R-ERK- NFκB axes induced cell proliferation by accelerating the G1–S transition.

To further determine the effect of the MC1R disruptive variants in these pathways, we overexpressed two common MC1R disruptive variants, R151C and R160W, in HEK 293 T cells and compared them with WT MC1R overexpressing HEK 293 T cells (Fig. 6d, Supplementary Fig. 6c). Stimulation with NDP-MSH increased ERK and p65 NFκB phosphorylation in the control HEK 293 T cells, but no increase in CREB phosphorylation was observed (Fig. 6d, Supplementary Fig. 6c). The WT MC1R-overexpressing HEK 293 T cells showed a significant increase in CREB, ERK, and p65 NFκB phosphorylation with NSP-MSH stimulation compared to the vector control HEK 293 T cells (Fig. 6d, Supplementary Fig. 6c). Interestingly, while NDP-MSH stimulation did increase CREB phosphorylation in the variant MC1R-overexpressing cells, this effect was significantly lower in the R160W MC1R-expressing cells (Fig. 6d, Supplementary Fig. 6c). On the other hand, no significant differences in ERK and p65 NFκB phosphorylation were observed between the three MC1R-expressing lines, although all three showed significantly increased ERK and p65 NFκB phosphorylation compared to the vector control HEK 293 T cells (Fig. 6d, Supplementary Fig. 6c). These results suggested different MC1R variants might affect MC1R downstream pathways and cell proliferation differently.

## Discussion

Expression of active *MC1R* variants in melanocytes is required for UV exposure-triggered synthesis of eumelanin, a photo-protective compound^3,28^. MC1R signaling also activates the DNA repair machinery and directs the repair of UV-induced DNA damage^7,35,36^. Additionally, MC1R signaling through the classical MC1R-PKA-CREB-MITF pathway decelerates cell proliferation^8^, although some studies have reported that αMSH can act as a weak mitogen for melanocyte proliferation in the presence of other growth promotors like basic fibroblast growth factor (bFGF) and tissue plasminogen activator (TPA)^9,11,12^. Together, these mechanisms are critical for preventing melanomagenesis. Although this function of MC1R is well characterized in melanocytes, MC1R’s functions in other cells, especially cells that do not produce pigment or those not exposed to light, are not well-studied. Given the anti-proliferative function of MC1R in melanocytes, its role in DNA repair, and its widespread expression in several cell types, we hypothesized that MC1R would have a protective effect on the pathogenesis of non-melanoma cancers. However, surprisingly, our preliminary analysis revealed a significant association between active MC1R germline variants and the development of different cancers (Fig. 1a). Our germline variant analysis demonstrated that relatively common *MC1R* variants associated with red hair and melanoma risk might unexpectedly be protective against the development of multiple cancers. We explored this association in breast cancer and discovered a significant relationship between *MC1R* expression and disease grade and progression (Fig. 1b–h, and 3e, f). We further characterized this new association in breast cancer and delineated the underlying mechanisms.

MC1R downregulation and inhibition decreased CREB/ATF-1, ERK, and p65 NFκB phosphorylation (Figs. 2e and 6a, b). CREB/ATF-1, ERK, and p65 NFκB are implicated in the regulation of Cyclin D1^34,37^, a key regulator of the G1 to S phase progression of the cell cycle. This suggested that MC1R signaling through the MC1R-cAMP-CREB/ATF-1 and MC1R-ERK-NFκB axes promotes cell cycle progression and accelerates cell proliferation in breast cancer cells. However, the detailed mechanisms underlying MC1R-induced growth in breast cancer cells need further confirmation. It remains unknown whether overactive MC1R or constitutive MC1R expression, by itself, are causative factors in breast cancer development. Nevertheless, our results suggest that active MC1R signaling could accelerate breast cancer progression. Since our analysis of the gene expression databases showed widespread *MC1R* expression in several tissue types and cancers, and our initial analysis showed a significant association between *MC1R* active variant frequency and several cancers, the effect of MC1R on other non-melanoma cancers should be further studied.

Our results showed that MC1R inhibition could significantly decrease cell proliferation in vitro (Fig. 2g–i). Further, MC1R-KD breast cancer cells formed significantly fewer colonies on soft agar (Fig. 3a, b) and significantly fewer tumors in athymic nude mice (Fig. 3c, d). This suggests the potential therapeutic effect of MC1R inhibition on breast cancer. However, further pre-clinical and animal studies should be conducted to explore the therapeutic effect of MC1R inhibition. Additionally, our analysis showed a significantly higher MC1R expression in breast cancer tissue than in adjacent normal tissue (Fig. 1b). Furthermore, TMA analysis showed a significant association between MC1R and Ki67, a proliferation marker (Fig. 3e, f). Finally, lower *MC1R* was associated with better disease-free and progression-free survival (Fig. 1c–h). Based on these results, the potential use of MC1R as a prognostic marker for breast cancer should be further explored. A previous study detected MC1R expression in 83% of the tested melanoma cell lines but not in other carcinoma lines, using immunohistochemistry^20^. However, we detected MC1R expression in breast cancer cell lines by western blot and in the breast cancer TMA by immunohistochemistry. This disparity might be due to the differences in cell lines and antibodies. Future studies on MC1R expression in different cancers, including breast cancer, must evaluate whether MC1R expression is sufficient for targeted therapy. We only determined the effect of total MC1R expression in our in vivo studies and TMA analysis, and *MC1R* variant-specific effects were only evaluated in HEK 293 T cells. Although an association between total MC1R expression and cell proliferation was observed, studies on the variant-specific effect on breast cancer proliferation are required for an in-depth understanding. Our results showed that HEK 293 T cells expressing the R151C and R160W MC1R variants showed varying degrees of CREB phosphorylation. However, both lines showed ERK phosphorylation comparable to the WT MC1R-expressing HEK 293 T cells. This aligns with previously published results^38^ and suggests that germline disruptive MC1R variants could also promote cell proliferation by signaling through the ERK-NFκB pathway, although signaling through the cAMP-CREB pathway might be dysfunctional. However, the physiological effect of the MC1R variants in promoting cell growth and cancer progression should be further evaluated in other models. Additionally, the MC1R-KD T-47d cells used in the study only have partial knockdown and not a complete knockout of MC1R. This condition could represent patients harboring germline *MC1R* R variants who show dysfunctional/disruptive but not complete loss of MC1R signaling. Interestingly, the MC1R-KD T-47d cells showed a much more pronounced decrease in tumorigenicity in vivo compared to the in vitro soft agar assay. This could plausibly be due to the continued αMSH stimulation in the in vivo condition.

In this study, NDP-MSH was used at a concentration of 0.2 μM for all experiments. Unfortunately, no studies have reported the level of αMSH in the breast tissue, although blood αMSH concentration has been reported to be around 10 pmol/l^39^. It has been reported that leptin in the adipose tissue might trigger the cleavage of proopiomelanocortin (POMC) to αMSH^40^, suggesting that the breast adipose tissue could be an alternate source of αMSH, which could trigger paracrine signaling within the breast tissue.

Interestingly, while treatment with NDP-MSH increased cell proliferation, MSG-606 not only attenuated the NDP-MSH-induced increase in cell proliferation but showed inhibitory effects on cell proliferation in both T-47d and MCF7 cells. No cell death or morphological changes were observed in cells treated with MSG-606 (data not shown). This suggests that MSG-606 has an inverse agonist effect on these cells. Further studies should explore the mechanisms underlying this inverse agonist effect of MSG-606. In this study, no significant changes in CREB or ERK phosphorylation were observed with lower (5 μM) concentrations of MSG-606, and a significant decrease in p65 NFκB phosphorylation was only observed with 10 μM MSG-606 (Fig. 6b, Supplementary Fig. 5b), suggesting that MSG-606 might have lower efficacy in breast cancer cells. Previous studies have shown a partial agonist effect of MSG-606 on other melanocortin receptors, MC3R and MC5R^30^. However, an analysis of the Human Protein Atlas database^23^ revealed that T-47d and MCF7 did not express MC3R and only had a very low expression of MC5R. Therefore, it is unlikely that the cells were stimulated through MC3R or MC5R.

A previous study showed that active MC1R and cAMP induction slowed the growth of melanoma cell lines by delaying G2/M progression caused by the increased inhibitory phosphorylation of cdc25B^8^. Further, they showed cAMP signaling inhibited MAPK signaling in NRas, but not BRaf mutant melanoma lines, without affecting S phase entry^8^. In contrast, our results show that MC1R activation accelerated S phase entry in breast cancer cells by promoting Cyclin D1 expression. These contrasting effects of MC1R activation in melanocytes and breast cancer might be due to the differences in downstream transcription factors. Although our results showed that MC1R signaled through CREB/ATF-1, ERK, and NFκB, the finer details and the specific transcription factors activated in different cell types should be identified in future studies.

Finally, several interventions to activate MC1R signaling^4,41^ and treatment with MC1R agonists^42^ are being developed or used in the clinic. However, their effect on the development of non-melanoma cancers is not well-studied, assuming that MC1R is primarily expressed in the melanocytes. Our results show widespread expression of MC1R across different tissues and, importantly, in different cancers. Further, since MC1R activation was shown to have growth-promoting effects, the tumorigenic potential of these MC1R-activating interventions should be re-evaluated, and such interventions should be used with caution.

In conclusion, our results showed that active MC1R signaling contributes to breast cancer progression in vitro and in vivo. Further characterization of this new association in breast cancer revealed that MC1R promotes breast cancer cell proliferation by signaling through the MC1R-cAMP-CREB/ATF-1 and MC1R-ERK-NFκB axes, resulting in accelerated progression of the cells from the G1 to the S phase of the cell cycle. Our results also suggested that MC1R could be developed as a prognostic marker for breast cancer. Additionally, since MC1R downregulation and inhibition decreased cell proliferation, MC1R inhibition should be evaluated as a potential treatment strategy for breast cancer. Finally, our results also suggested that MC1R may be involved in the development of multiple cancers other than melanoma and plays relevant protective roles in a tissue-specific manner.

## Methods

### Experimental model details

#### Animal studies

The animal experiment protocol was approved by the Institutional Animal Care and Use Committee of the Cleveland Clinic (Approval No. 00002768) and was in accordance with the Animal Welfare Act (AWA) and Public Health Service (PHS) Policy of the United States of America. Female BALB/c athymic nude (Nu/J) mice (8 weeks old) (RRID: IMSR_JAX:002019) purchased from Jackson Laboratories were used for the study. The mice were housed in groups, of not more than five, in plastic cages with stainless-steel grid tops in a barrier unit within the animal care facility of the Cleveland Clinic Lerner Research Center with water and food supplied *ad libitum* in a 12 h light/dark cycle. At 10 weeks of age, the mice were randomly grouped into the Control and Test groups (*n* = 7 in each group).

#### Cell lines

HEK 293 T cells and human breast cancer cell lines T-47d and MCF7 were obtained from the American Type Culture (ATCC) and were maintained in high glucose Dulbecco’s modified Eagle’s medium (DMEM; Invitrogen; Cat. #11995073), supplemented with 10% fetal bovine serum (FBS; Invitrogen; Cat. #26140079) and antibiotic–antimycotic mixture (Invitrogen; Cat. #15240096) (100 units/ml) at 37 °C and 5% CO_2_.

### Bioinformatics analysis

To study the effect of germline *MC1R* variants on different cancer types, we extracted *MC1R* germline mutations from the whole-exome sequences of 10,391 cancer patients housed in TCGA (phs000178.v11.p8) and an independent breast cancer dataset (Breast Cancer Genome Guided Therapy Study (BEAUTY), phs001050.v1.p1, *N* = 110). Our inclusion criteria for notable disruptive *MC1R* R alleles were defined according to the criteria outlined previously^5^, and the variants are listed in Supplementary Table 1. These disruptive R allele mutations are characterized to disable the function of normal MC1R signaling. Inclusion was limited to patients listed as white individuals, as *MC1R* R variants are significantly more prevalent in the Caucasian population. The frequency of disruptive R allele variants was calculated across cancer types in TCGA and the BEAUTY study. The total and allelic frequencies of patients with any R allele for each dataset (*R*% count by allele) were determined. The Exome Aggregation Consortium (ExAC) Non-TCGA Non-Finnish European Ancestry (ExAC Non-TCGA NFE) population was used to estimate a control frequency of R variants using the same criteria for the R alleles above. This was used to match the ethnicity category used in our cancer analysis. The ExAC Non-TCGA NFE was used as the control population as it included healthy people of European ancestry and as the R alleles are rare in the non-European population. Additionally, the Finnish population was not included in the control population because of the high frequency of founder mutations in this population. Fisher’s exact analysis was performed for each cancer type against this general population to calculate the difference in *R*% allele counts. Multiple testing correction was done with Benjamini–Hochberg (BH) multiple comparisons adjustment.

### MC1R knockdown (MC1R-KD) and overexpression

For generating stable MC1R knockdown T-47d cell lines, MC1R shRNA-carrying lentiviral particles were produced by transient transfection of HEK 293 T cells with a VSV-G envelope expressing plasmid, pMD2.G (a gift from Didier Trono; Addgene plasmid # 12259; http://n2t.net/addgene:12259; RRID: Addgene_12259), a 2^nd^ generation lentiviral packaging plasmid, pCMVR8.74 (a gift from Didier Trono; Addgene plasmid # 22036; http://n2t.net/addgene:22036; RRID: Addgene_22036), and either MC1R shRNA TRCN0000357545 (Thermo Fisher) (targeting CAACCTCTTTCTCGCCCTCAT; Sh1) or TRCN0000357609 (Thermo Fisher) (targeting TGCGGCTGCATCTTCAAGAAC; Sh2), using Lipofectamine 3000 (Thermo Fisher; Cat. #13778075) transfection reagent, following the manufacturer’s protocol. Lentiviral supernatants were collected 24 h and 48 h after transfection and stored at −80 °C until use. T-47d cells were cultured in fresh culture media containing 8 μg/ml polybrene (Santa Cruz; Cat. #sc-134220) and infected with the lentiviral supernatant. Stable MC1R-KD cells were selected by culturing the cells with 2.5 μg/ml puromycin (Invivogen; Cat. #ant-pr-1) for 4 days. The stable cell lines were maintained in complete DMEM, and cell stocks were prepared and stored in liquid nitrogen.

For transient MC1R overexpression, HEK 293 T cells, plated in 6-well plates in complete DMEM, were transfected with an MC1R overexpression plasmid (OriGene; Cat#: RC203218) using Lipofectamine 3000 (Thermo Fisher). The MC1R variant plasmids (R151C and R160W) were a kind gift from Dr. Shuyang Chen.

### Cell growth and tumorigenicity

To determine cell growth, 15,000 cells in 100 μl media were plated in each well of a 96-well plate and incubated at 37 °C. After overnight incubation, the cells were rinsed with PBS, serum-starved (DMEM + 0.5% FBS) for 10 h, and then returned to fresh media with or without 0.2 μM NDP-MSH (Tocris; Cat. #3013) or 20 μM MSG-606 (Tocris; Cat. #59541). The initial cell number (Time 0) was determined at 6 h post-treatment. At the indicated time points, 10 μl WST-8 solution (Colorimetric Cell Viability Kit 1, PromoKine; Cat. #PK-CA705-CK04) was added to each well, and the plates were incubated at 37 °C for 45 min. After incubation, the absorbance at 450 nm was read using a Varioskan LUX multimode microplate reader (Thermo Fisher). The average value from triplicates was used for the calculations.

To evaluate tumorigenicity, 2500 cells were suspended in 0.35% agarose-DMEM and layered on 0.5% agar-DMEM in 6-well plates. Complete media (1 ml) was layered over the agarose layer, and the cells were cultured in a humidified incubator at 37 °C and 5% CO_2_. Fresh media was added to the wells every 3 days. After 3 weeks, the gels were stained with crystal violet (Sigma-Aldrich; Cat. #HT90132-1L), and the plates were observed under a wide field microscope (Leica DM5500B, Leica Microsystems, GmbH, Wetzlar, Germany), and the images were acquired using a Leica DFC425C color camera (Leica Microsystems, GmbH, Wetzlar, Germany) and the LASX software. The number of colonies in each well was counted using Image-Pro Plus (Media Cybernetics, Inc., Rockville, MD). The average from triplicate wells was used for calculations, and control wells with no cells were used to eliminate the background.

### cAMP assay

Cells cultured in 96-well tissue culture plates were serum-starved (DMEM + 0.5% FBS) for 10 h and then treated with fresh serum-starvation media supplemented with 0.2 μM NDP-MSH. After 15 m, the cAMP levels were determined using the Cyclic AMP XP Assay Kit (Cell Signaling Technology; Cat. #4339), following the manufacturer’s protocol. For evaluating cAMP synthesis with MC1R inhibition, the cells were pretreated with 20 μM MSG-606 for 3 h before being stimulated with 0.2 μM NDP-MSH. For evaluating cAMP synthesis with forskolin (FSK; Research Product International; Cat. # F20685) stimulation, the cells were treated with 25 μM FSK after serum starvation.

### Cell cycle progression analysis

The cells were synchronized to the G1/S phase using a double-thymidine block. Briefly, cells were grown in complete DMEM to approximately 40% confluency, treated with 2 mM thymidine (Sigma-Aldrich; Cat. #T1895) for 16 h, washed with PBS, and grown in complete DMEM for 9 h. They were then returned to media containing 2 mM thymidine for 14 h, and then rinsed with PBS and released to thymidine-free media with or without various treatments (0.2 μM ND-MSH, 20 μM MSG-606, 25 μM forskolin, or 0.2 μM NDP-MSH + 5 μM 666-15 (Millipore Sigma; Cat. #5383410001)). All cell cycle progression experiments were performed using serum-starvation/low-serum conditions. At 0, 3, 6, 9, and 12 h post-release from the double-thymidine block, the cells were collected and fixed in cold 70% ethanol. The cells were then stained with 10 μg/ml propidium iodide (Sigma-Aldrich; Cat. #P4864) with RNaseA (Millipore Sigma; Cat. #70856-3) for 30 min in the dark at room temperature and immediately placed on ice. Fluorescence data were acquired on a BD Fortessa (BD Biosciences) and analyzed using ModFit LT 6.0 (Verity Software House). All cell cycle progression experiments were carried out in triplicates; the mean values from the triplicates are shown in the results.

### Western blot

Total cell lysates were prepared using M-per Mammalian Protein Extraction Reagent (Thermo Fisher; Cat. #78501) containing protease and phosphatase inhibitors (Halt Protease and Phosphatase Inhibitor Cocktail (100X) (Thermo Fisher; Cat. #78440)). Equal amounts of proteins were resolved by SDS-PAGE, transferred to nitrocellulose membranes (Bio-Rad; Cat. #1620115), blocked with 5% milk or EveryBlot Blocking Buffer (Bio-Rad; Cat. #12010020) for 1 h, and incubated with primary antibodies (overnight at 4 °C, after which they were washed and incubated with corresponding HRP-conjugated secondary antibodies. The primary antibodies used in the study were anti-MC1R (Invitrogen; Cat. #PA5-97961; 1:1000), CREB (86B10) Mouse mAb (Cell Signaling; Cat. #9104; 1:1000), Cyclin D1 (Cell Signaling; Cat. #2922; 1:1500), Cyclin E1 (Cell Signaling; Cat. #4129; 1:2000), phospho-Rb (Ser780) (Cell Signaling; Cat. #8180; 1:1000), Rb (Cell Signaling; Cat. #9309: 1:2000), ERK 1/2 (C-9) (Santa Cruz; Cat. #sc-514302; 1:1000), GAPDH (14C10) (Cell Signaling; Cat. #2118; 1:10000), NF-κB p65 (93H1) (Cell Signaling; Cat. #8242; 1:1000), Phospho-CREB (Ser133) (87G3) Rabbit mAb (Cell Signaling; Cat. #9198; 1:1000), Phospho-NF-κB p65 (Ser536) (Cell Signaling; Cat. #3033; 1:1000), Phospho-p44/42 MAPK (ERK 1/2) (Thr202/Tyr204) XP Rabbit mAb (Cell Signaling; Cat. #4370: 1:1000); and the secondary antibodies used were Anti-Mouse IgG Secondary HRP Conjugate (Promega, Cat. #W402B; 1:2500) and Anti-Rabbit Secondary IgG HRP Conjugate (Promega, Cat. #W401B; 1:2500). The protein bands were developed using the ECL Western Blotting Substrate (Bio-Rad; Cat. #32106) and visualized using a ChemiDoc imager (Bio-Rad). The band densities were quantified using ImageJ, and the relative protein amounts were calculated by normalizing to the corresponding loading control band. All uncropped blots are included in the Source Data file.

### In vivo tumor growth assay

Female BALB/c athymic nude (Nu/J) mice were purchased from Jackson Laboratories and housed in a barrier unit within the animal care facility of the Cleveland Clinic Lerner Research Center. At 10 weeks of age, the mice were randomly grouped into two groups (Control and Test; *n* = 7 in each group), and all mice were implanted with a 0.36 mg 17β-estradiol 30-day release pellet (Innovative Research of America; Cat. #SE-121) under the skin behind their necks using a sterile trocar. At the same time, T-47d and MC1R-KD T-47d cells (1 × 10^6^ cells) prepared in sterile normal saline were injected subcutaneously into the right hind flanks of the mice in the Control and Test groups, respectively. The mice were examined every day for the development of tumors and other adverse events. After the development of palpable tumors, they were measured in two dimensions using a digital Vernier caliper every other day. Tumor volume was calculated using the formula *V* = (*W*^2^ × *L*)/2, where *W* is the width (diameter perpendicular to the largest diameter), and *L* is the length (largest diameter).

### Tumor microarray and immunohistochemistry

Breast cancer tumor microarray, BC081116e (TissueArray.com LLC; Cat. #, BC081116e) containing 110 cores with 107 cases representing breast cancer with cancer-adjacent breast tissue array, including invasive carcinoma of no special type, breast carcinoma with apocrine differentiation, and AT tissue was purchased from TissueArray.com LLC, and the associated data, including pathology grade, IHC (ER/PR/Her-2/Ki67) information, and TNM/Stage(AJCC 7th edition) was downloaded. According to TissueArray.com LLC, all tissues were collected under the highest ethical standards with the donor being informed completely and with their consent; steps were taken to ensure that standard medical care was followed and the donors’ privacy was protected; and all human tissues were collected under Health Insurance Portability and Accountability Act (HIPAA)-approved protocols.

Immunohistochemistry staining was performed using the Discovery ULTRA automated stainer (Roche Diagnostics). In brief, antigen retrieval was performed using a tris/borate/EDTA buffer (Discovery CC1; Roche; Cat. #06414575001), pH 8.0 to 8.5, for 32 min at 95 °C, followed by incubation with 1:200 diluted anti-MC1R antibody (Invitrogen; Cat. #PA5-97961), for 1 h at room temperature. The antibody was visualized using the OmniMap anti-Rabbit HRP (Roche; Cat. #05269679001) in conjunction with the ChromoMap DAB detection kit (Roche; Cat. #05266645001), followed by counterstaining with hematoxylin and bluing. The TMA was scanned at 40× using an Aperio AT2 slide scanner (Leica Biosystems), and the images were viewed and exported as .tiff images using QuPath^43^. The images were scored based on percentage staining and staining intensity by a pathologist blinded to the tissue samples. The scoring scheme was as follows: Percentage staining score 0, no staining; 1, <25%; 2, 25–50%, 3, 50–75%, 4, 75–100%; Staining intensity score 0, no staining; 1, mild staining, 2, moderate staining, 3, high staining. The final score was calculated as the product of the two scores (staining score = percentage staining × staining intensity).

### Statistical analysis

All statistical analyses were performed using GraphPad Prism Ver 9.4 (GraphPad Software, La Jolla, CA, USA) R (version 4.0.3), and RStudio (version 1.3.1093). The specific tests used for each analysis are described in the Figure legends. A *p*-value < 0.05 was considered statistically significant.

### Reporting summary

Further information on research design is available in the Nature Research Reporting Summary linked to this article.

### Supplementary information

Reporting Summary

Supplementary Information

## Data Availability

The authors confirm that the data supporting the findings of this study are available within the article and/or its supplementary materials. The Cancer Genome Atlas (TCGA; dbGaP Study Accession: phs000178.v11.p8) and Breast Cancer Genome Guided Therapy Study (BEAUTY; dbGaP Study Accession phs001050.v1.p1) datasets were retrieved through the database of Genotypes and Phenotypes (dbGaP) under authorization to YN (Project no. 21943). The Exome Aggregation Consortium (ExAC) Non-TCGA Non-Finnish European Ancestry dataset is available from https://gnomad.broadinstitute.org/downloads#exac. The bulk tissue gene expression of MC1R in human tissue data is openly available from the GTEx portal (dbGaP Study Accession phs000424.v8.p2). The MC1R expression in cell lines data is available from the Broad CCLE Portal (https://depmap.org/portal/download/all/) and the Human Protein Atlas (https://www.proteinatlas.org/ENSG00000258839-MC1R/cell+line). The TMA data are available from TissueArray.com LLC (Cat. No: BC081116e; https://www.tissuearray.com/tissue-arrays/Breast/BC081116e).
