# Supplementary material for: MC1R signaling through the cAMP-CREB/ATF-1 and ERK-NFκB pathways accelerates G1/S transition promoting breast cancer progression
Source: NPJ Precis Oncol. 2023 Sep 7;7:85. doi: 10.1038/s41698-023-00437-1 (PMC10485002; doi:10.1038/s41698-023-00437-1)
Supplement: Supplementary file 1 — Reporting Summary [file 41698_2023_437_MOESM1_ESM.pdf]

# nature portfolio

Corresponding author(s): Joshua Arbesman, Ying Ni

Last updated by author(s): Aug 7, 2023

# Reporting Summary

Nature Portfolio wishes to improve the reproducibility of the work that we publish. This form provides structure for consistency and transparency in reporting. For further information on Nature Portfolio policies, see our Editorial Policies and the Editorial Policy Checklist.

## Statistics

For all statistical analyses, confirm that the following items are present in the figure legend, table legend, main text, or Methods section.

| n/a | Confirmed | |
|---|---|---|
| ☐ | ☒ | The exact sample size (*n*) for each experimental group/condition, given as a discrete number and unit of measurement |
| ☐ | ☒ | A statement on whether measurements were taken from distinct samples or whether the same sample was measured repeatedly |
| ☐ | ☒ | The statistical test(s) used AND whether they are one- or two-sided<br>*Only common tests should be described solely by name; describe more complex techniques in the Methods section.* |
| ☒ | ☐ | A description of all covariates tested |
| ☐ | ☒ | A description of any assumptions or corrections, such as tests of normality and adjustment for multiple comparisons |
| ☐ | ☒ | A full description of the statistical parameters including central tendency (e.g. means) or other basic estimates (e.g. regression coefficient) AND variation (e.g. standard deviation) or associated estimates of uncertainty (e.g. confidence intervals) |
| ☒ | ☐ | For null hypothesis testing, the test statistic (e.g. *F*, *t*, *r*) with confidence intervals, effect sizes, degrees of freedom and *P* value noted<br>*Give P values as exact values whenever suitable.* |
| ☒ | ☐ | For Bayesian analysis, information on the choice of priors and Markov chain Monte Carlo settings |
| ☒ | ☐ | For hierarchical and complex designs, identification of the appropriate level for tests and full reporting of outcomes |
| ☒ | ☐ | Estimates of effect sizes (e.g. Cohen's *d*, Pearson's *r*), indicating how they were calculated |

*Our web collection on statistics for biologists contains articles on many of the points above.*

## Software and code

Policy information about availability of computer code

| | |
|---|---|
| Data collection | No software used |
| Data analysis | GraphPad Prism Ver 9.4 (GraphPad Software, La Jolla, CA, USA), R (version 4.0.3), RStudio (version 1.3.1093) |

For manuscripts utilizing custom algorithms or software that are central to the research but not yet described in published literature, software must be made available to editors and reviewers. We strongly encourage code deposition in a community repository (e.g. GitHub). See the Nature Portfolio guidelines for submitting code & software for further information.

## Data

Policy information about availability of data

All manuscripts must include a data availability statement. This statement should provide the following information, where applicable:

- Accession codes, unique identifiers, or web links for publicly available datasets
- A description of any restrictions on data availability
- For clinical datasets or third party data, please ensure that the statement adheres to our policy

The authors confirm that the data supporting the findings of this study are available within the article and/or its supplementary materials.

## Research involving human participants, their data, or biological material

Policy information about studies with human participants or human data. See also policy information about sex, gender (identity/presentation), and sexual orientation and race, ethnicity and racism.

| | |
|---|---|
| Reporting on sex and gender | *Use the terms sex (biological attribute) and gender (shaped by social and cultural circumstances) carefully in order to avoid confusing both terms. Indicate if findings apply to only one sex or gender; describe whether sex and gender were considered in study design; whether sex and/or gender was determined based on self-reporting or assigned and methods used.*<br>*Provide in the source data disaggregated sex and gender data, where this information has been collected, and if consent has been obtained for sharing of individual-level data; provide overall numbers in this Reporting Summary. Please state if this information has not been collected.*<br>*Report sex- and gender-based analyses where performed, justify reasons for lack of sex- and gender-based analysis.* |
| Reporting on race, ethnicity, or other socially relevant groupings | *Please specify the socially constructed or socially relevant categorization variable(s) used in your manuscript and explain why they were used. Please note that such variables should not be used as proxies for other socially constructed/relevant variables (for example, race or ethnicity should not be used as a proxy for socioeconomic status).*<br>*Provide clear definitions of the relevant terms used, how they were provided (by the participants/respondents, the researchers, or third parties), and the method(s) used to classify people into the different categories (e.g. self-report, census or administrative data, social media data, etc.)*<br>*Please provide details about how you controlled for confounding variables in your analyses.* |
| Population characteristics | *Describe the covariate-relevant population characteristics of the human research participants (e.g. age, genotypic information, past and current diagnosis and treatment categories). If you filled out the behavioural & social sciences study design questions and have nothing to add here, write "See above."* |
| Recruitment | *Describe how participants were recruited. Outline any potential self-selection bias or other biases that may be present and how these are likely to impact results.* |
| Ethics oversight | *Identify the organization(s) that approved the study protocol.* |

Note that full information on the approval of the study protocol must also be provided in the manuscript.

# Field-specific reporting

Please select the one below that is the best fit for your research. If you are not sure, read the appropriate sections before making your selection.

☒ Life sciences ☐ Behavioural & social sciences ☐ Ecological, evolutionary & environmental sciences

For a reference copy of the document with all sections, see nature.com/documents/nr-reporting-summary-flat.pdf

# Life sciences study design

All studies must disclose on these points even when the disclosure is negative.

| | |
|---|---|
| Sample size | Based on previous studies (Proc Natl Acad Sci U S A. 2005 Sep 27;102(39):14034-9, Clin Cancer Res. 2001 Oct;7(10):3156-65) we anticipate that the control (T47D WT) cells would form a tumor that is ~10 cm in diameter in about 3 weeks. Assuming that 90% of the mice would develop a tumor in the WT (control) group and and 20% would develop a tumor in the test group (based on in vitro experiments), using Lamorte's Power Calculations, we determined that, we would need 7 mice per group for 95% probability of showing a statistically significant difference (using $p< 0.05$). |
| Data exclusions | *Describe any data exclusions. If no data were excluded from the analyses, state so OR if data were excluded, describe the exclusions and the rationale behind them, indicating whether exclusion criteria were pre-established.* |
| Replication | *Describe the measures taken to verify the reproducibility of the experimental findings. If all attempts at replication were successful, confirm this OR if there are any findings that were not replicated or cannot be reproduced, note this and describe why.* |
| Randomization | The mice were randomly assigned to the study groups. |
| Blinding | Animal studies: The person measuring the tumors were blinded to the study groups.<br>TMA: The pathologist scoring the TMA was blinded to the sample set. |

# Reporting for specific materials, systems and methods

We require information from authors about some types of materials, experimental systems and methods used in many studies. Here, indicate whether each material, system or method listed is relevant to your study. If you are not sure if a list item applies to your research, read the appropriate section before selecting a response.

## Materials & experimental systems

| n/a | Involved in the study |
|---|---|
| ☐ | ☒ Antibodies |
| ☐ | ☒ Eukaryotic cell lines |
| ☒ | ☐ Palaeontology and archaeology |
| ☐ | ☒ Animals and other organisms |
| ☒ | ☐ Clinical data |
| ☒ | ☐ Dual use research of concern |
| ☒ | ☐ Plants |

## Methods

| n/a | Involved in the study |
|---|---|
| ☒ | ☐ ChIP-seq |
| ☐ | ☒ Flow cytometry |
| ☒ | ☐ MRI-based neuroimaging |

# Antibodies

Antibodies used

The primary antibodies used in the study were anti-MC1R (Invitrogen; Cat. #PA5-97961), CREB (86B10) Mouse mAb (Cell Signaling; Cat. #9104), Cyclin D1 (Cell Signaling; Cat. #2922), Cyclin E1 (Cell Signaling; Cat. #4129), phospho-Rb (Ser780) (Cell Signaling; Cat. #8180), Rb (Cell Signaling; Cat. #9309), ERK 1/2 (C-9) (Santa Cruz; Cat. #sc-514302), GAPDH (14C10) (Cell Signaling; Cat. #2118), NF-κB p65 (93H1) (Cell Signaling; Cat. #8242), Phospho-CREB (Ser133) (87G3) Rabbit mAb (Cell Signaling; Cat. #9198), Phospho-NF-κB p65 (Ser536) (Cell Signaling; Cat. #3033), Phospho-p44/42 MAPK (ERK 1/2) (Thr202/Tyr204) XP Rabbit mAb (Cell Signaling; Cat. #4370); and the secondary antibodies used were Anti-Mouse IgG Secondary HRP Conjugate (Promega, Cat. #W402B) and Anti-Rabbit Secondary IgG HRP Conjugate (Promega, Cat. #W401B).

Validation

MC1R-Invitrogen; Cat. #PA5-97961: The antibody was affinity-purified from rabbit antiserum by affinity-chromatography using epitope-specific immunogen and the purity is > 95% (by SDS-PAGE). Validated for WB, ICC/IF, and IHC.

CREB (86B10) CST #9104: Monoclonal antibody is produced by immunizing animals with recombinant protein specific to human CREB-1 protein. Specificity / Sensitivity:  CREB (86B10) Mouse mAb detects endogenous levels of total CREB-1 protein. The antibody may also detect the CREB-related protein, ATF-1. Species Reactivity: Human, Mouse, Rat, Monkey, Validated for WB, IF, IHC

Cyclin D1 Antibody CST #2922: Specificity / Sensitivity: Cyclin D1 Antibody detects endogenous levels of cyclin D1. It does not cross-react with other family members. Species Reactivity: Human. Validated for WB, IP

Cyclin E1 (HE12) Mouse mAb CST #4129: Specificity / Sensitivity: Cyclin E1 (HE12) Mouse mAb detects endogenous levels of total cyclin E1 protein. It does not cross react with cyclin E2. Species Reactivity: Human, Monkey. Validated for WB.

Phospho-Rb (Ser780) (D59B7) Rabbit mAb CST #8180: Specificity / Sensitivity: Phospho-Rb (Ser780) (D59B7) Rabbit mAb recognizes endogenous levels of Rb protein only when phosphorylated at Ser780. Species Reactivity: Human, Mouse, Rat, Monkey. Validated for WB, IP.

Rb (4H1) Mouse mAb CST #9309: Specificity / Sensitivity: Rb (4H1) Mouse mAb detects endogenous levels of total Rb protein. The antibody does not cross-react with the Rb homologues p107 or p130, or with other proteins. Species Reactivity: Human, Monkey, Bovine, Pig. Validated for WB, IP, IHC, IF, FC, ChIP.

ERK 1/2 Antibody (C-9): sc-514302: ERK 1/2 (C-9) is a mouse monoclonal antibody raised against amino acids 101-172 mapping near the N-terminus of ERK 2 of human origin. ERK 1/2 (C-9) is recommended for detection of ERK 1 and ERK 2 of mouse, rat and human origin by Western Blotting (starting dilution 1:100, dilution range 1:100-1:1000), immunoprecipitation [1-2 µg per 100-500 µg of total protein (1 ml of cell lysate)], immunofluorescence (starting dilution 1:50, dilution range 1:50-1:500), immunohistochemistry (including paraffin-embedded sections) (starting dilution 1:50, dilution range 1:50-1:500) and solid phase ELISA (starting dilution 1:30, dilution range 1:30-1:3000). Molecular Weight of ERK 1: 44 kDa. Molecular Weight of ERK 2: 42 kDa. Positive Controls: DU 145 cell lysate: sc-2268, Jurkat whole cell lysate: sc-2204 or K-562 whole cell lysate: sc-2203.

GAPDH (14C10) Rabbit mAb CST #2118: Specificity / Sensitivity: GAPDH (14C10) Rabbit mAb detects endogenous levels of total GAPDH protein. Species Reactivity: Human, Mouse, Rat, Monkey, Bovine, Pig. Species predicted to react based on 100% sequence homology: Pig. Validated for WB, IHC, IC, FC.

NF-κB p65 (D14E12) XP® Rabbit mAb CST #8242: Specificity / Sensitivity: NF-κB p65 (D14E12) XP® Rabbit mAb recognizes endogenous levels of total NF-κB p65/RelA protein. It does not cross react with other NF-κB/Rel family members. Species Reactivity: Human, Mouse, Rat, Hamster, Monkey, Dog. Validated for WB, IP, IHC, IF, FC, ChIP, ChIP-seq, CUT&RUN.

Phospho-CREB (Ser133) (87G3) Rabbit mAb CST #9198: pecificity / Sensitivity: Phospho-CREB (Ser133) (87G3) Rabbit mAb detects endogenous levels of CREB only when phosphorylated at serine 133. The antibody also detects the phosphorylated form of the CREB-related protein, ATF-1. Species Reactivity: Human, Mouse, Rat. Species predicted to react based on 100% sequence homology: Zebrafish. Validated for WB, IHC, IF, FC, ChIP, ChIP-seq, CUT&RUN.

Phospho-NF-κB p65 (Ser536) (93H1) Rabbit mAb CST #3033: Specificity / Sensitivity: Phospho-NF-kappaB p65 (Ser536) (93H1) Rabbit mAb detects NF-κB p65 only when phosphorylated at Ser536. It does not cross-react with the p50 subunit or other related proteins. Species Reactivity: Human, Mouse, Rat, Hamster, Monkey, Pig. Species predicted to react based on 100% sequence homology: Dog. Validated for WB, IP, IF, FC.

Phospho-p44/42 MAPK (Erk1/2) (Thr202/Tyr204) (D13.14.4E) XP® Rabbit mAb CST #4370: Specificity / Sensitivity Phospho-p44/42 MAPK (Erk1/2) (Thr202/Tyr204) (D13.14.4E) XP® Rabbit mAb detects endogenous levels of p44 and p42 MAP Kinase (Erk1 and Erk2) when dually phosphorylated at Thr202 and Tyr204 of Erk1 (Thr185 and Tyr187 of Erk2), and singly phosphorylated at

Thr202. This antibody does not cross-react with the corresponding phosphorylated residues of either JNK/SAPK or p38 MAP kinases. Species Reactivity: Human, Mouse, Rat, Hamster, Monkey, Mink, D. melanogaster, Zebrafish, Bovine, Dog, Pig, S. cerevisiae. Species predicted to react based on 100% sequence homology: Chicken, C. elegans. Validated for WB, IP, IFC, FC.

# Eukaryotic cell lines

Policy information about cell lines and Sex and Gender in Research

| | |
|---|---|
| Cell line source(s) | All cell lines were purchased from the ATCC. |
| Authentication | Further authentication was not performed. |
| Mycoplasma contamination | All cell lines used in the study tested negative for mycoplasma contamination. |
| Commonly misidentified lines (See ICLAC register) | N/A |

# Animals and other research organisms

Policy information about studies involving animals; ARRIVE guidelines recommended for reporting animal research, and Sex and Gender in Research

| | |
|---|---|
| Laboratory animals | Female BALB/c athymic nude (Nu/J) mice (8 weeks old) (RRID: IMSR_JAX:002019) purchased from Jackson Laboratories were used for the study. |
| Wild animals | N/A |
| Reporting on sex | Female mice were used in the study. |
| Field-collected samples | N/A |
| Ethics oversight | The animal experiment protocol was approved by the Institutional Animal Care and Use Committee of the Cleveland Clinic (Approval No. 00002768) and was in accordance with the Animal Welfare Act (AWA) and Public Health Service (PHS) Policy of the United States of America. |

Note that full information on the approval of the study protocol must also be provided in the manuscript.

# Flow Cytometry

## Plots

Confirm that:

☒ The axis labels state the marker and fluorochrome used (e.g. CD4-FITC).

☒ The axis scales are clearly visible. Include numbers along axes only for bottom left plot of group (a 'group' is an analysis of identical markers).

☒ All plots are contour plots with outliers or pseudocolor plots.

☒ A numerical value for number of cells or percentage (with statistics) is provided.

## Methodology

| | |
|---|---|
| Sample preparation | Cells were collected and fixed in cold 70% ethanol. The cells were then stained with 10 µg/ml propidium iodide (Sigma-Aldrich; Cat. #P4864-10mL ) with RNaseA (Millipore Sigma; Cat. #70856-3) for 30 min in the dark at room temperature and immediately placed on ice. |
| Instrument | BD Fortessa |
| Software | Data acquisition: BD FACSDiva, Data analysis: ModFit LT 6.0 |
| Cell population abundance | Cells were not sorted. |
| Gating strategy | Cells were first gated with the FSC-Area and SSC-Area plot, and further gated to remove doublets with propidium iodide height vs propidium iodide width plot, where singlets are clustered vertically. |

☒ Tick this box to confirm that a figure exemplifying the gating strategy is provided in the Supplementary Information.
