# Supplementary material for: MC1R signaling through the cAMP-CREB/ATF-1 and ERK-NFκB pathways accelerates G1/S transition promoting breast cancer progression
Source: NPJ Precis Oncol. 2023 Sep 7;7:85. doi: 10.1038/s41698-023-00437-1 (PMC10485002; doi:10.1038/s41698-023-00437-1)

Supplementary Information

MC1R signaling through the cAMP-CREB/ATF-1 and ERK-NFκB pathways accelerates G1/S transition promoting breast cancer progression

Vipin Shankar Chelakkot[1], Kiara Thomas[1], Todd Romigh[1], Andrew Fong[1], Lin Li[2], Shira Ronen[3], Shuyang Chen[1], Pauline Funchain[4], Ying Ni[2]*, Joshua Arbesman[1,5,6] *

[1]Department of Cancer Biology, Lerner Research Institute, Cleveland Clinic, Cleveland, Ohio, USA

[2]Center for Immunotherapy & Precision Immuno-Oncology, Lerner Research Institute, Cleveland Clinic, Cleveland Clinic, Cleveland, Ohio, USA

[3]Department of Anatomic Pathology, Pathology and Laboratory Medicine Institute, Cleveland Clinic, Cleveland, Ohio, USA

[4]Department of Hematology & Oncology, Taussig Cancer Center, Cleveland Clinic, Cleveland, Ohio, USA

[5]Department of Dermatology, Dermatology and Plastic Surgery Institute, Cleveland Clinic, Cleveland, Ohio, USA

[6]Department of Dermatology, Cleveland Clinic Lerner College of Medicine, Case Western Reserve University, Cleveland, Ohio, USA

***Corresponding Authors**

Joshua Arbesman: arbesmj@ccf.org; Ying Ni: niy@ccf.org

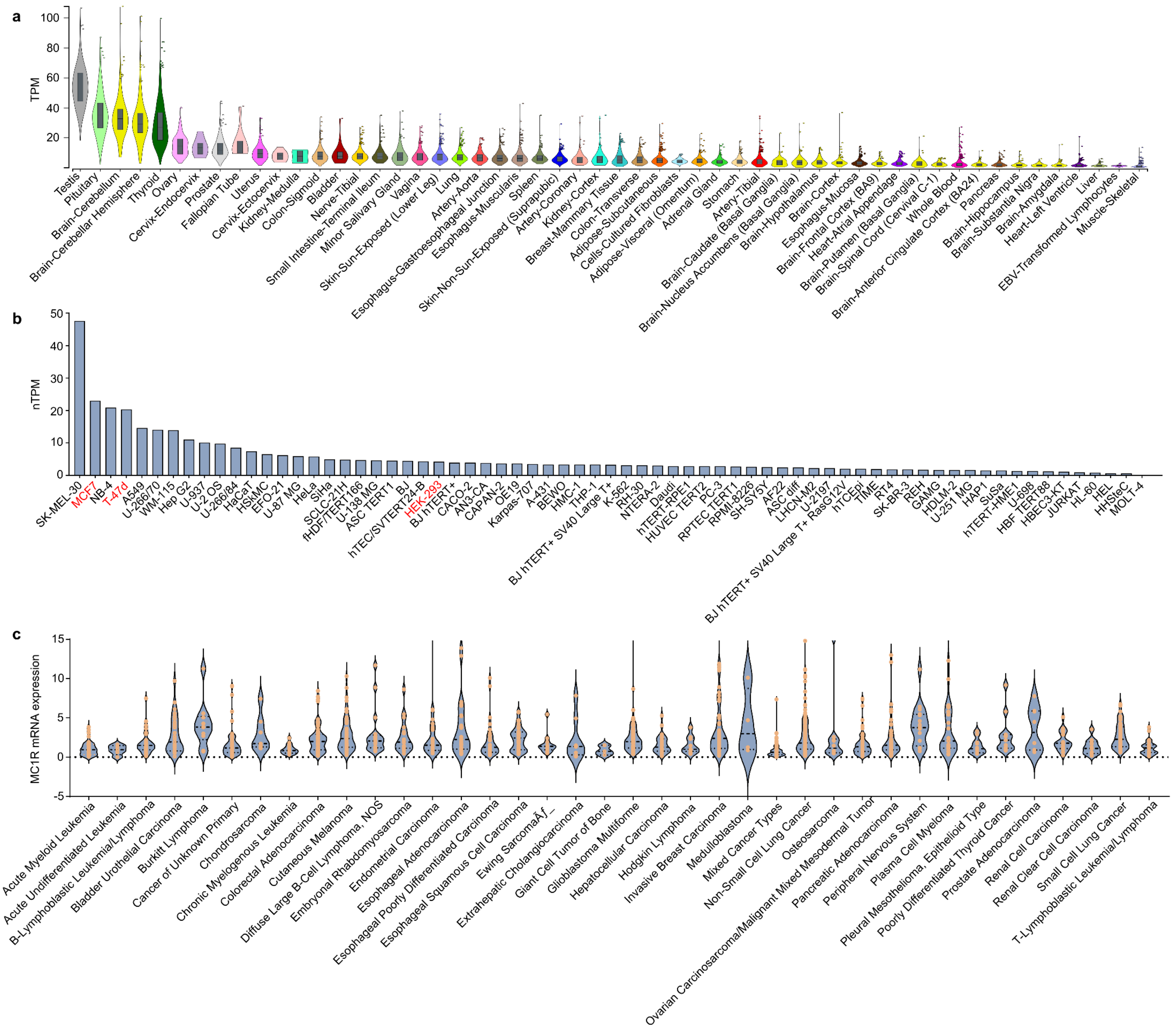
a
TPM
100
80
60
40
20
Testis
Pituitary
Brain-Cerebellum
Brain-Cerebellar Hemisphere
Thyroid
Ovary
Cervix-Endocervix
Prostate
Fallopian Tube
Uterus
Cervix-Ectocervix
Kidney-Medulla
Colon-Sigmoid
Bladder
Nerve-Tibial
Small Intestine-Terminal Ileum
Minor Salivary Gland
Vagina
Skin-Sun-Exposed (Lower Leg)
Lung
Artery-Aorta
Esophagus-Gastroesophageal Junction
Esophagus-Muscularis
Spleen
Skin-Non-Sun-Exposed (Suprapubic)
Artery-Coronary
Kidney-Cortex
Breast-Mammary Tissue
Colon-Transverse
Adipose-Subcutaneous
Cells-Cultured Fibroblasts
Adipose-Visceral (Omentum)
Adrenal Gland
Stomach
Artery-Tibial
Brain-Caudate (Basal Ganglia)
Brain-Nucleus Accumbens (Basal Ganglia)
Brain-Hypothalamus
Brain-Cortex
Esophagus-Mucosa
Brain-Frontal Cortex (BA9)
Heart-Atrial Appendage
Brain-Putamen (Basal Ganglia)
Brain-Spinal Cord (Cervival C-1)
Whole Blood
Brain-Anterior Cingulate Cortex (BA24)
Pancreas
Brain-Hippocampus
Brain-Substantia Nigra
Brain-Amygdala
Heart-Left Ventricle
Liver
EBV-Transformed Lymphocytes
Muscle-Skeletal
b
nTPM
50
40
30
20
10
0
SK-MEL-30
MCF7
NB-4
T-47d
A549
U-266/70
WM-115
Hep G2
U-937
U-2 OS
U-266/84
HaCaT
HSKMC
EFO-21
U-87 MG
HeLa
SiHa
SCLC-21H
fHDF/TERT166
U-138 MG
ASC TERT1
BJ
hTEC/SVTERT24-B
HEK-293
BJ hTERT+
CACO-2
AN3-CA
CAPAN-2
OE19
Karpas-707
A-431
BEWO
HMC-1
THP-1
BJ hTERT+ SV40 Large T+
K-562
RH-30
NTERA-2
Daudi
hTERT-RPE1
HUVEC TERT2
PC-3
RPTEC TERT1
RPMI-8226
SH-SY5Y
AF22
ASC diff
LHCN-M2
U-2197
BJ hTERT+ SV40 Large T+ RasG12V
hTCEpi
TIME
RT4
SK-BR-3
REH
GAMG
HDLM-2
U-251 MG
HAP1
SuSa
hTERT-HME1
U-698
HBF TERT88
HBEC3-KT
JURKAT
HL-60
HEL
HHSteC
MOLT-4
c
MC1R mRNA expression
15
10
5
0
-5
Acute Myeloid Leukemia
Acute Undifferentiated Leukemia
B-Lymphoblastic Leukemia/Lymphoma
Bladder Urothelial Carcinoma
Burkitt Lymphoma
Cancer of Unknown Primary
Chondrosarcoma
Chronic Myelogenous Leukemia
Colorectal Adenocarcinoma
Cutaneous Melanoma
Diffuse Large B-Cell Lymphoma, NOS
Embryonal Rhabdomyosarcoma
Endometrial Carcinoma
Esophageal Adenocarcinoma
Esophageal Poorly Differentiated Carcinoma
Esophageal Squamous Cell Carcinoma
Ewing SarcomaÃƒ_
Extrahepatic Cholangiocarcinoma
Giant Cell Tumor of Bone
Glioblastoma Multiforme
Hepatocellular Carcinoma
Hodgkin Lymphoma
Invasive Breast Carcinoma
Medulloblastoma
Mixed Cancer Types
Non-Small Cell Lung Cancer
Osteosarcoma
Ovarian Carcinosarcoma/Malignant Mixed Mesodermal Tumor
Pancreatic Adenocarcinoma
Peripheral Nervous System
Plasma Cell Myeloma
Pleural Mesothelioma, Epithelioid Type
Poorly Differentiated Thyroid Cancer
Prostate Adenocarcinoma
Renal Cell Carcinoma
Renal Clear Cell Carcinoma
Small Cell Lung Cancer
T-Lymphoblastic Leukemia/Lymphoma

Supplementary Figure 1. *MC1R* is widely expressed in human tissues and human cancer cell lines

a. Bulk tissue gene expression of *MC1R* (ENSG00000258839.3) in human tissues. Data Source: GTEx Analysis Release V8 (dbGaP Accession phs000424.v8.p2). Expression values are shown in TPM (Transcripts Per Million), calculated from a gene model with isoforms collapsed to a single gene. No other normalization steps have been applied. Box plots show the median and 25th and 75th percentiles; points are displayed as outliers if they are above or below 1.5 times the interquartile range. The testis, brain, thyroid, and ovary showed high expression, while the expression in the skin (sun-exposed lower leg) was much lower.

b, c. *MC1R* expression in cancer cell lines **(b)** data from The Human Protein Atlas **(c)** data from the cancer cell encyclopedia analyzed using cBioPortal. nTPM: normalized Transcripts Per Million. The dashed lines in the violin plot show the median and the 25th and 75th percentiles. The cell lines used in this study are indicated using red font in **b**.

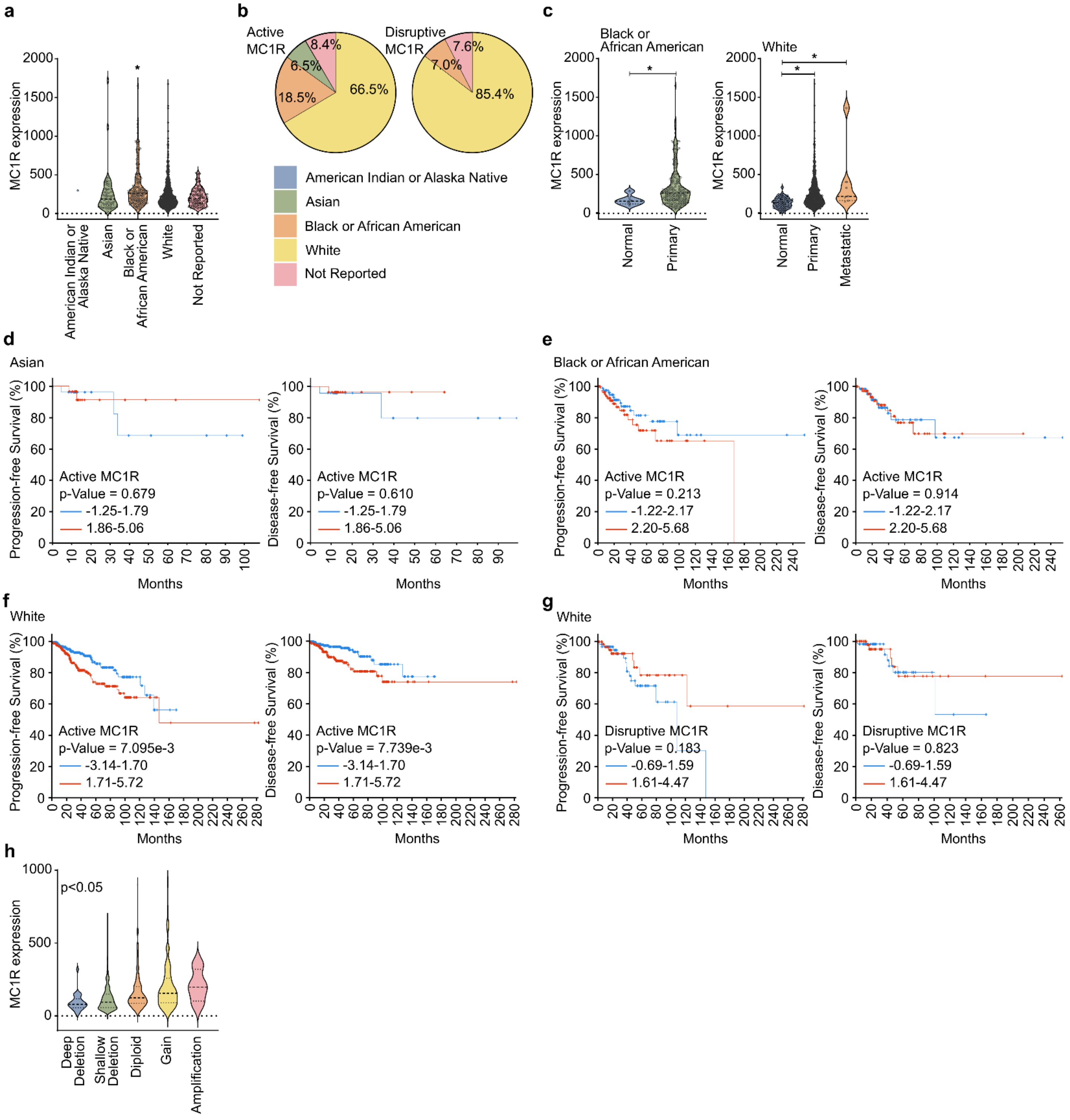

Supplementary Figure 2.

a. Expression of *MC1R* (RSEM, Batch normalized from Illumina HiSeq_RNASeqV2; log2) among people of different ethnicities (Data from TCGA). The dashed lines in the violin plot show the median and the 25th and 75th percentiles. *p<0.05 (one-way ANOVA with Dunnett's multiple comparisons).

b. Distribution of active *MC1R* and disruptive *MC1R* variants among people of different ethnicities. *$p<0.05$ (one-way ANOVA with Dunnett's multiple comparisons).

c. *MC1R* mRNA expression (RSEM, Batch normalized from Illumina HiSeq_RNASeqV2; log2) in normal breast, primary, and metastatic breast cancers among the Black or African American and white people. The dashed lines in the violin plot show the median and the 25th and 75th percentiles. Data obtained from TCGA. *$p<0.05$ (Student's *t*-test or one-way ANOVA with Dunnett's multiple comparisons).

d–g. Progression-free survival (PFS) and Disease-free survival (DFS) of (**d**) Asian, (**e**) Black or African American, and (**f**) white people carrying active MC1R and (**g**) white people carrying disruptive MC1R variants with breast cancer. p-Value calculated using the Log-rank test.

h. Violin plot comparing MC1R expression (Batch normalized from Illumina HiSeq_RNASeqV2) (log2(value+1)) and putative copy-number alterations. The dashed lines in the violin plot show the median and the 25th and 75th percentiles. p-Value calculated using one-way ANOVA.

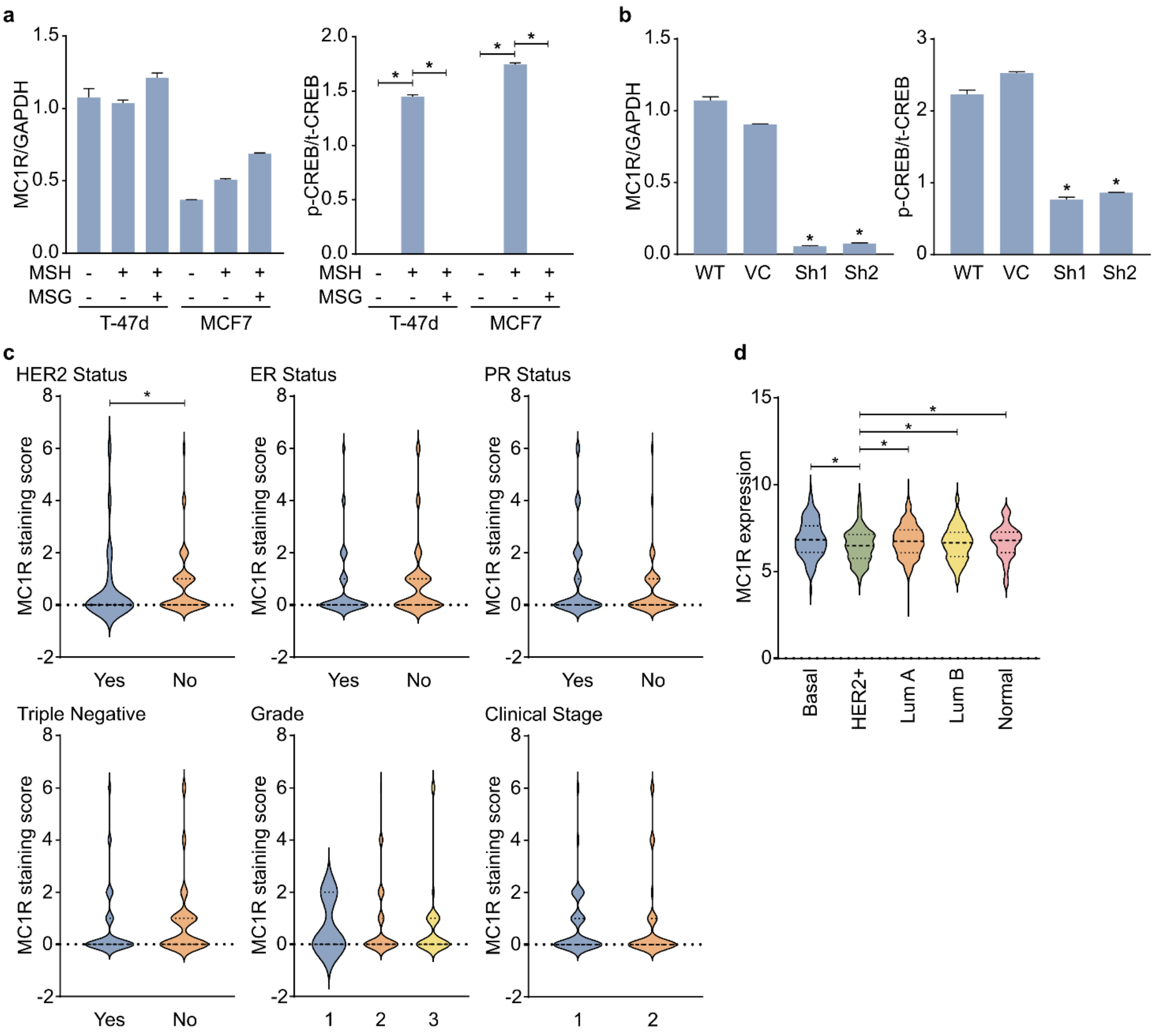

Supplementary Figure 3. a, b. Quantification of western blots shown in (**a**) Figure 2d and (**b**) Figure 2e. Data shows mean ± SEM values from three independent experiments. *p<0.0-5 (one-way ANOVA with Tukey's multiple comparisons).

c. Plots comparing MC1R staining score with HER2 status, ER status, PR status, Triple Negative status, Grade, and the clinical stage (1= IIA or below, local/primary disease; 2= IIB and above, local or distant metastasis) of breast cancer tissue in the tumor microarray. A significant association between HER2-negative breast cancers and high MC1R staining was observed. The dashed lines in the violin plot show the median and the 25th and 75th percentiles. *p<0.05 unpaired Student's *t*-test.

d. Plot comparing *MC1R* mRNA expression (RSEM, Batch normalized from Illumina HiSeq_RNASeqV2; log2) with the breast cancer types. The dashed lines in the violin plot show the median and the 25th and 75th percentiles. Data from TCGA. (NA: Type not available) *p<0.05, one-way ANOVA with Dunnett's multiple comparisons).

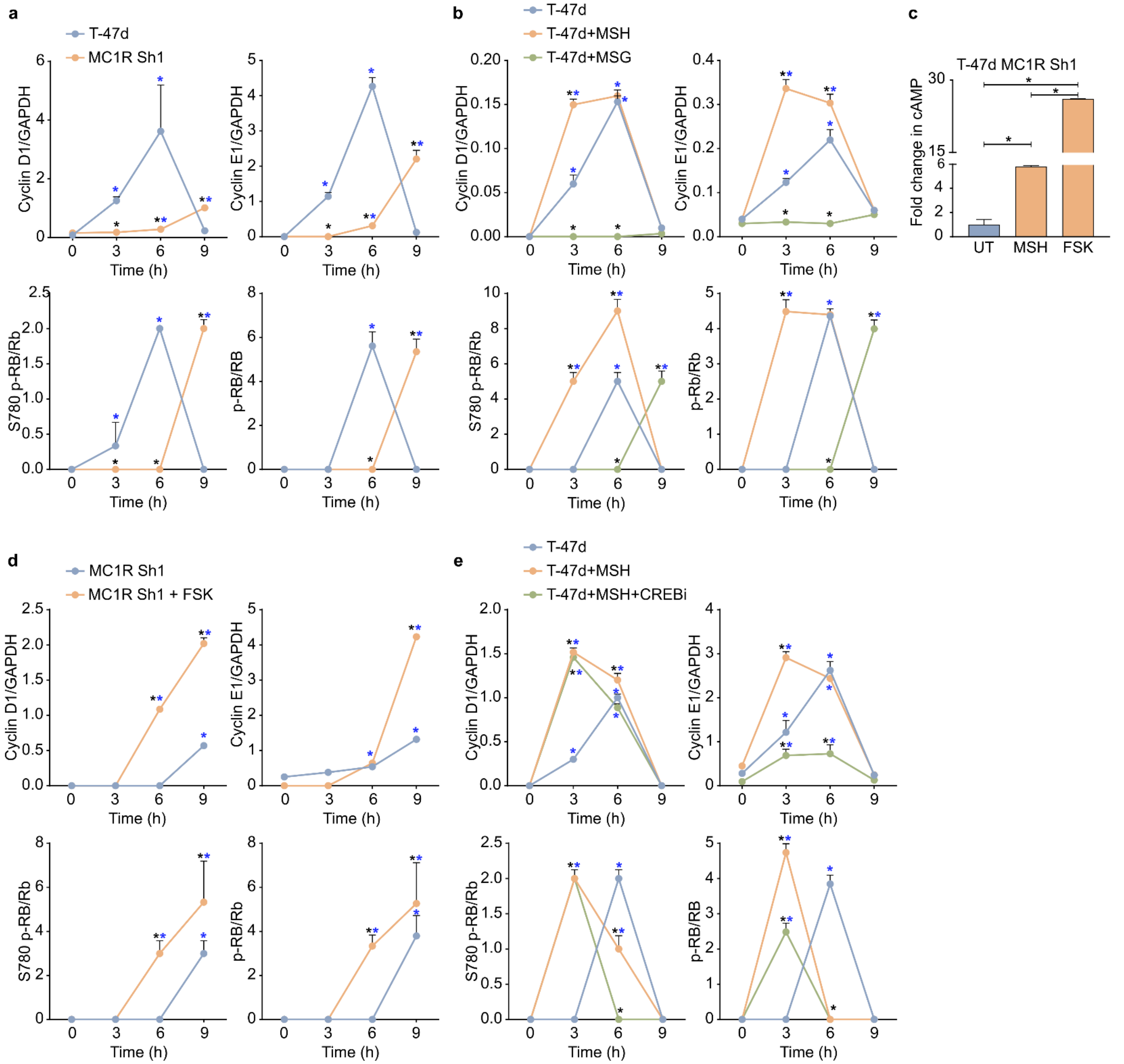

Supplementary Figure 4.

a, b. Quantification of western blot shown in (**a**) Figure 4c and (b) Figure 4f. Data shows mean ± SEM values from three independent experiments. Black * denotes p<0.0-5 (two-way ANOVA with Dunnett's multiple comparisons) compared to T-47d at the respective time points. Blue * denotes p<0.05 (two-way ANOVA with Dunnett's multiple comparisons) compared to time 0 within the same cell line/treatment condition.

c. The MC1R KD-T-47d cells were treated with 0.2 μM NDP-MSH or 25 μM FSK or left untreated. Mean ± SEM percentage fold change in cAMP levels from 3 independent experiments. *p<0.05 (one-way ANOVA with Tukey's multiple comparisons).

d, e. Quantification of western blot shown in (**d**) Figure 5c and (e) Figure 5f. Data shows mean ± SEM values from three independent experiments. Black * denotes p<0.0-5 (two-way ANOVA with Dunnett's multiple comparisons) compared to the control (T-47d MC1R Sh1 in d and T-47d in e) at the respective time points. Blue * denotes p<0.05 (two-way ANOVA with Dunnett's multiple comparisons) compared to time 0 within the same cell line/treatment condition.

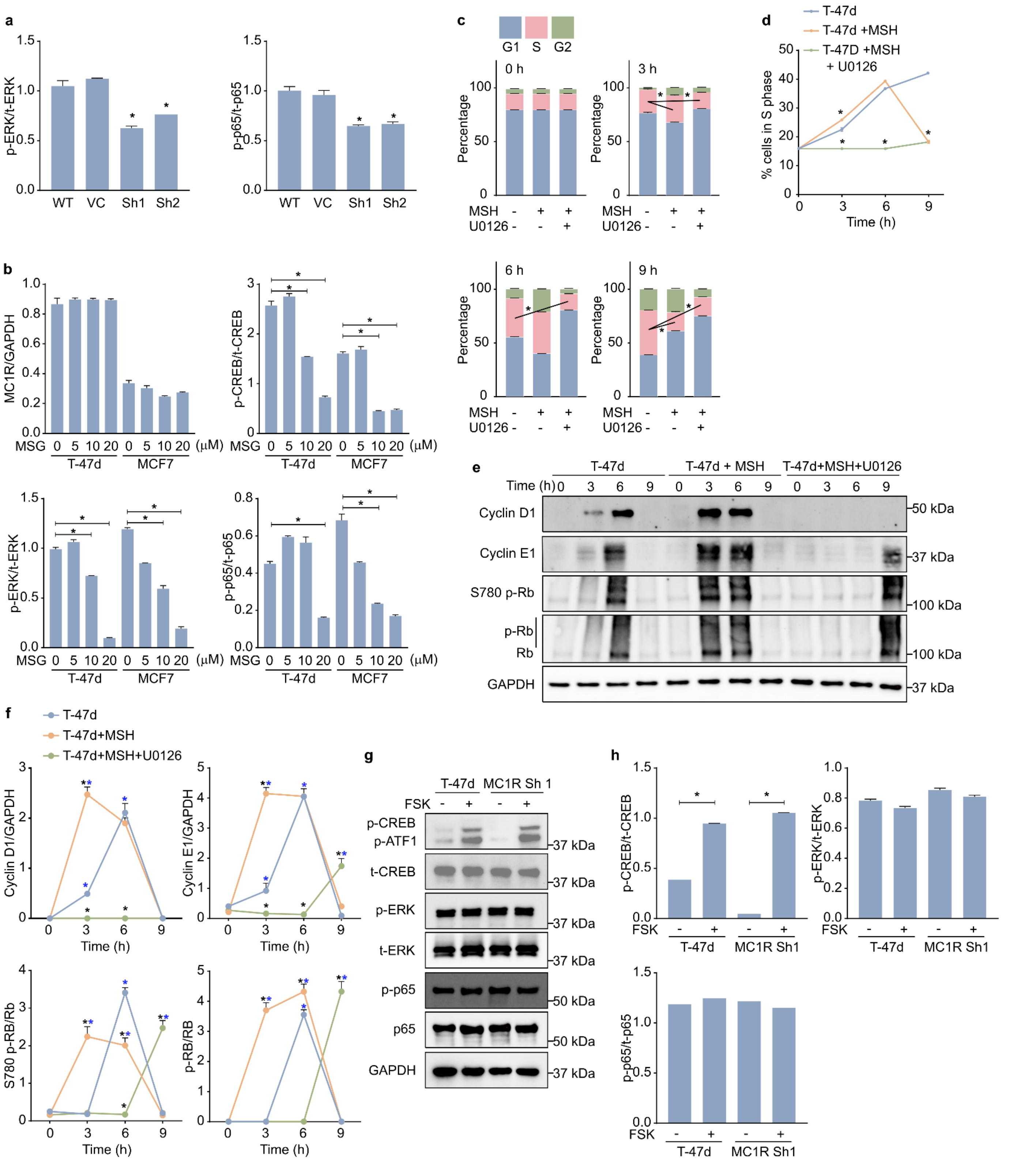

Supplementary Figure 5.

a, b. Quantification of western blot shown in (**a**) Figure 6a and (**b**) Figure 6b. Data shows mean ± SEM values from three independent experiments. * p<0.0-5 (one-way ANOVA with Tukey’s multiple comparisons).

c–f. WT T-47d cells were treated with 0.2 μM NDP-MSH or 0.2 μM NDP-MSH + 5 μM U0126 or left untreated after releasing from a double-thymidine block. (**c**) Mean ± SEM percentage of cells in the G1, S, and G2 phases at 0, 3, 6, and 9, post-release. *$p<0.05$ (unpaired Student's t-test). (**d**) Mean ± SEM percentage of cells in the S phase across time. *$p<0.05$ (one-way ANOVA with Dunnett's multiple comparisons). (**e**) Representative western blot and (**f**) quantification plots showing Cyclin D1, Cyclin E1, S780 pRB, Rb, and GAPDH (loading control). Data shows mean ± SEM values from three independent experiments. Black * denotes p<0.0-5 (two-way ANOVA with Dunnett's multiple comparisons) compared to T-47d at the respective time points. Blue * denotes $p<0.05$ (two-way ANOVA with Dunnett's multiple comparisons) compared to time 0 within the same treatment condition.

g–h. WT T-47d and MC1R knockdown (KD) T-47d cells were treated with or without 25 μM FSK. (**g**) Representative western and (**h**) quantification plots blot showing p-CREB, p-ATF1, t-CREB, p-ERK, t-ERK, p-p65 NFκB, p65 NFκB, and GAPDH (loading control). Data shows mean ± SEM values from three independent experiments. * p<0.0-5 (one-way ANOVA with Tukey's multiple comparisons).

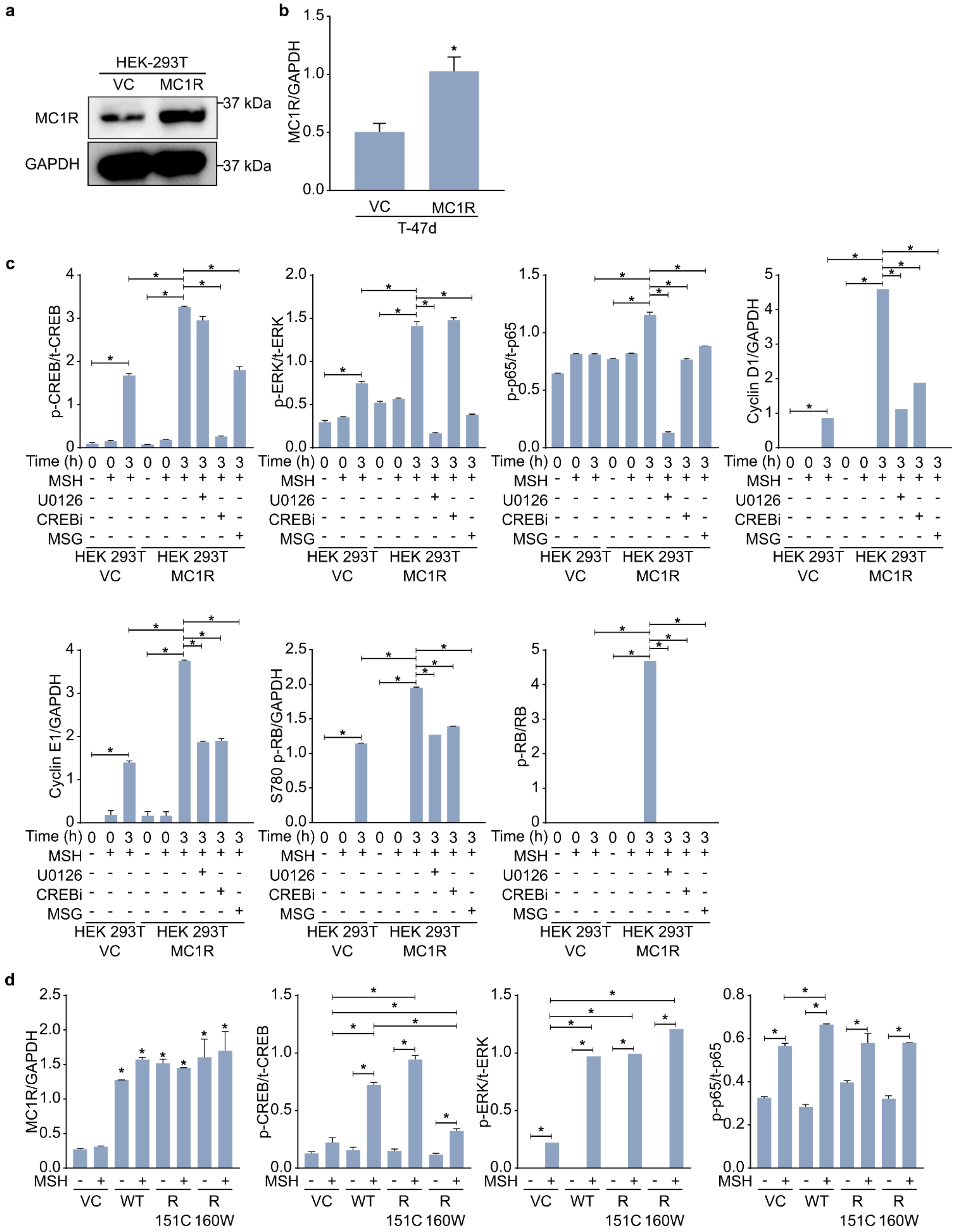

Supplementary Figure 6.

a, b. (**a**) Representative western blot and (**b**) quantification plot showing transient overexpression of MC1R in HEK-293T cells transfected with an MC1R plasmid. GAPDH expression is shown as the loading control. VC: vector control.

c, d. Quantification of western blot shown in (**c**) Figure 6c and (**d**) Figure 6d. Data shows mean ± SEM values from three independent experiments. * p<0.0-5 (one-way ANOVA with Tukey's multiple comparisons). VC: vector control.

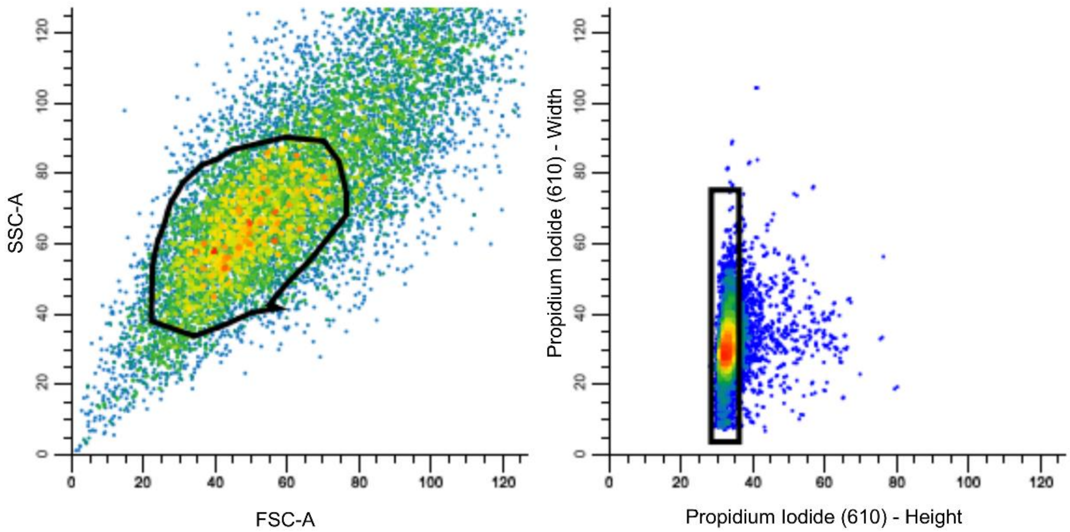

Supplementary Figure 7. Gating strategy for cell cycle progression flow cytometry experiments.

Data were acquired on a BD Fortessa (BD Biosciences) and analyzed using ModFit LT 6.0 (Verity Software House). Cells were first gated with the FSC-Area and SSC-Area plot and further gated to remove doublets with propidium iodide height vs propidium iodide width plot, where singlets are clustered vertically.

MC1R R (Disruptive variant)	p.R	Mutation
D84E	p.D84E	16:89985918 C / A (rs1805006)
R142H	p.R142H	16:89986091 G / A (rs11547464)
R151C	p.R151C	16:89986117 C / T (rs1805007)
I155T	p.I155T	16:89986130 T / C (rs1110400)
R160W	p.R160W	16:89986144 C / T (rs1805008)
D294H	p.D294H	16:89986546 G / C (rs1805009)
Y152X	p.Y152X	16:89986122 C / A
S83P	p.S83P	16:89985913 T / C (rs34474212)
G89R	p.G89R	16:89985931 G / A (rs34540312)
T95M	p.T95M	16:89985950 C / T (rs34158934)
D121E	p.D121E	16:89986029 C / G
A149T	p.A149T	16:89986111 G / A
V156L	p.V156L	16:89986132 G / C (rs3212365)
V156A	p.V156A	16:89986133 T / C
I168M	p.I168M	16:89986170 C / G (rs34612847)
R306H	p.R306H	16:89986583 G / A
Q23X	p.Q23X	16:89985733 C / T
Y74X	p.Y74X	16:89985888 C / A
Y207X	p.Y207X	16:89986287 C / G
A28fs	p.A28fs	16:89985750 dupA
F179fs	p.F179fs	16:89986202 T / TC
A212fs	p.A212fs	16:89986301 delCCCGGGCCTGCCAGCACGC
I221fs	p.I221fs	16:89986329 delCGCCCGGCTCCACAAGAGGCAG
R229fs	p.R229fs	16:89986350 GC / G
L237fs	p.L237fs	16:89986377 dupA

Supplementary Table 1. List of disruptive MC1R (R) variants.

Cancer Type	Abbreviation	R% count by allele	Total Allele	R Allele	Non-R Allele	Odd's Ratio (OR)	OR. Lower CI	OR. Upper CI	p-value (Fisher's Exact Test)	Adjusted p-value (BH)
Uterine Carcinosarcoma	UCS	0.2955	88	26	62	1.7158	1.0410	2.7550	0.0305	0.0837
Skin Cutaneous Melanoma	SKCM	0.2864	894	256	638	1.6418	1.4121	1.9042	1.72E-10	5.67E-09
Rectum Adenocarcinoma	READ	0.2375	160	38	122	1.2744	0.8606	1.8491	0.1953	0.4027
Kidney Renal Papillary Cell Carcinoma	KIRP	0.2181	408	89	319	1.1416	0.8912	1.4498	0.2874	0.5042
Pancreatic Adenocarcinoma	PAAD	0.2063	320	66	254	1.0632	0.7976	1.4002	0.6719	0.8623
Bladder Urothelial Carcinoma	BLCA	0.2034	654	133	521	1.0445	0.8557	1.2676	0.6558	0.8623
Head & Neck Squamous Cell Carcinoma	HNSC	0.2030	872	177	695	1.0420	0.8772	1.2326	0.6363	0.8623
Liver Hepatocellular Carcinoma	LIHC	0.2016	372	75	297	1.0332	0.7903	1.3366	0.7934	0.8788
Prostate Adenocarcinoma	PRAD	0.2007	294	59	235	1.0273	0.7581	1.3731	0.8255	0.8788
Mesothelioma	MESO	0.2000	160	32	128	1.0229	0.6712	1.5179	0.9206	0.9415
**ExAC Non-TCGA, Non-Finnish European Ancestry (ExACNon-TCGA NFE) Control Population**		**0.1963**								
Pheochromocytoma and Paraganglioma	PCPG	0.1926	296	57	239	0.9758	0.7171	1.3088	0.9415	0.9415
Brain Lower Grade Glioma	LGG	0.1907	944	180	764	0.9640	0.8137	1.1372	0.6794	0.8623
Uterine Corpus Endometrial Carcinoma	UCEC	0.1905	740	141	599	0.9631	0.7949	1.1606	0.7442	0.8788
Lung Adenocarcinoma	LUAD	0.1872	780	146	634	0.9422	0.7805	1.1313	0.5548	0.8623
Acute Myeloid Leukemia	LAML	0.1865	252	47	205	0.9381	0.6677	1.2945	0.7507	0.8788
Kidney Chromophobe	KICH	0.1810	116	21	95	0.9045	0.5350	1.4634	0.8148	0.8788

Lung Squamous Cell Carcinoma	LUSC	0.1796	696	125	571	0.8957	0.7312	1.0902	0.2903	0.5042
Colon Adenocarcinoma	COAD	0.1745	424	74	350	0.8651	0.6632	1.1157	0.2696	0.5042
Cholangiocarcinoma	CHOL	0.1711	76	13	63	0.8443	0.4261	1.5506	0.6659	0.8623
Thymoma	THYM	0.1667	204	34	170	0.8183	0.5484	1.1891	0.3310	0.5461
Kidney Renal Clear Cell Carcinoma	KIRC	0.1661	548	91	457	0.8148	0.6426	1.0235	0.0832	0.2112
Sarcoma	SARC	0.1518	448	68	380	0.7322	0.5566	0.9509	0.0168	0.0503
Thyroid Carcinoma	THCA	0.1492	650	97	553	0.7177	0.5717	0.8931	0.0024	0.0098
Breast Invasive Carcinoma	BRCA	0.1490	1430	213	1217	0.7161	0.6151	0.8305	4.75E-06	5.23E-05
Adrenocortical Carcinoma	ACC	0.1474	156	23	133	0.7076	0.4334	1.1079	0.1307	0.2875
Stomach Adenocarcinoma	STAD	0.1426	554	79	475	0.6805	0.5288	0.8660	0.0012	0.0057
Glioblastoma Multiforme	GBM	0.1395	674	94	580	0.6631	0.5269	0.8269	0.0001	0.0010
Uveal Melanoma	UVM	0.1364	110	15	95	0.6461	0.3478	1.1207	0.1193	0.2812
Testicular Germ Cell Tumors	TGCT	0.1303	238	31	207	0.6128	0.4056	0.8974	0.0088	0.0321
Cervical Squamous Cell Carcinoma and Endocervical Adenocarcinoma	CESC	0.1292	418	54	364	0.6070	0.4470	0.8103	0.0004	0.0022
Ovarian Serous Cystadenocarcinoma	OV	0.1279	688	88	600	0.6001	0.4737	0.7525	3.11E-06	5.13E-05
Esophageal Carcinoma	ESCA	0.1009	228	23	205	0.4591	0.2844	0.7085	0.0001	0.0010
Lymphoid Neoplasm Diffuse Large B-cell Lymphoma	DLBC	0.0500	40	2	38	0.2154	0.0252	0.8335	0.0158	0.0503

Supplementary Table 2. **R% allele frequency among 10,391 patients with cancer housed in TCGA grouped based on 33 cancer types**. The cancer types, their R% allele frequencies, and the p-values compared to the control population determined by Fisher's exact test and Benjamini–Hochberg (BH) multiple comparison adjustment are shown. The cancer types are listed in the decreasing order of their R% allele frequencies. The R% allele frequency of the control Exome Aggregation Consortium (ExAC) Non-TCGA, Non-Finnish European Ancestry (ExACNon-TCGA NFE) population was 0.1963 (Indicated using orange font in the table). Skin Cutaneous Melanoma with a significantly higher R% allele frequency than the control population is indicated using red font, and cancers with significantly lower R% allele frequencies than the control population are indicated using blue font. Of the nine cancers that showed a significantly different R% allele frequency compared to the control population, eight cancer types (thyroid carcinoma, breast invasive carcinoma, stomach adenocarcinoma, glioblastoma multiforme, testicular germ cell tumors, cervical squamous cell carcinoma and endocervical adenocarcinoma, ovarian serous cystadenocarcinoma, and esophageal carcinoma) showed significantly lower R% allele frequencies. Breast Invasive Carcinoma (highlighted in orange in the table), which showed one of the lowest p-values (p=5.23E-05), was chosen for further exploration of the association between MC1R and cancer progression.

Genotype	0,0	0,r	R,0	r,r	R,r	R,R	Total	RR% count by individual	R% count by Allele	Total Allele	No. of R	No. of non-R	p-Value
**Frequency**	43	29	18	7	9	4	110	0.03636364	0.15909091	220	35	185	0.32

Supplementary Table 3. Analysis of R% allele frequency in the Breast Cancer Genome Guided Therapy Study (BEAUTY) (dbGaP accession phs001050.v1.p1) dataset of patients with breast cancer. The R% allele frequency was 0.1591, which is lower than that of the control population (0.1963), although not statistically significant (p-value = 0.32, Chi-squared test)

Figure 2d. T-47d and MCF7 MC1R-CREB signaling

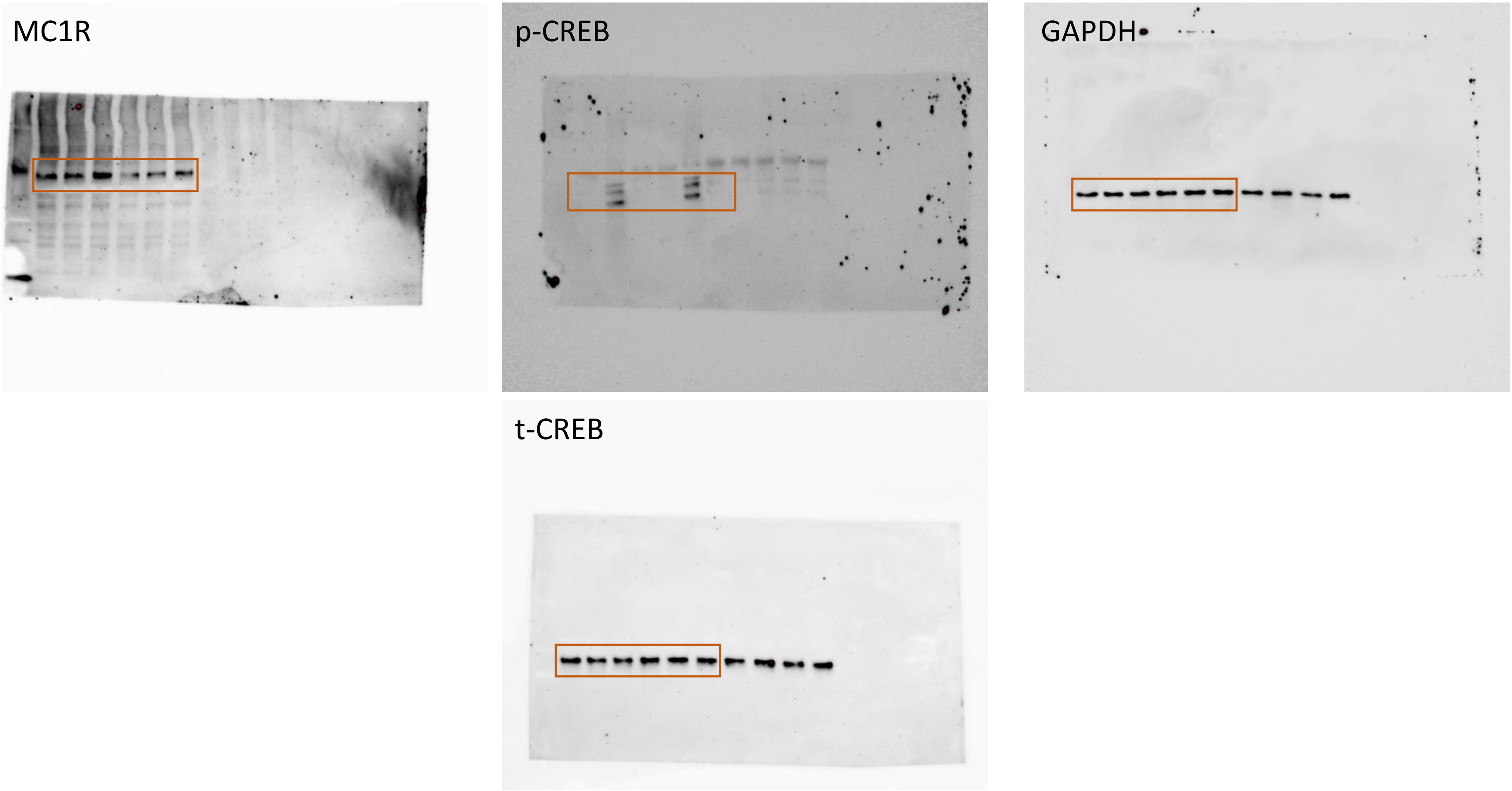

Figure 2e. T-47d MC1R KD CREB signaling

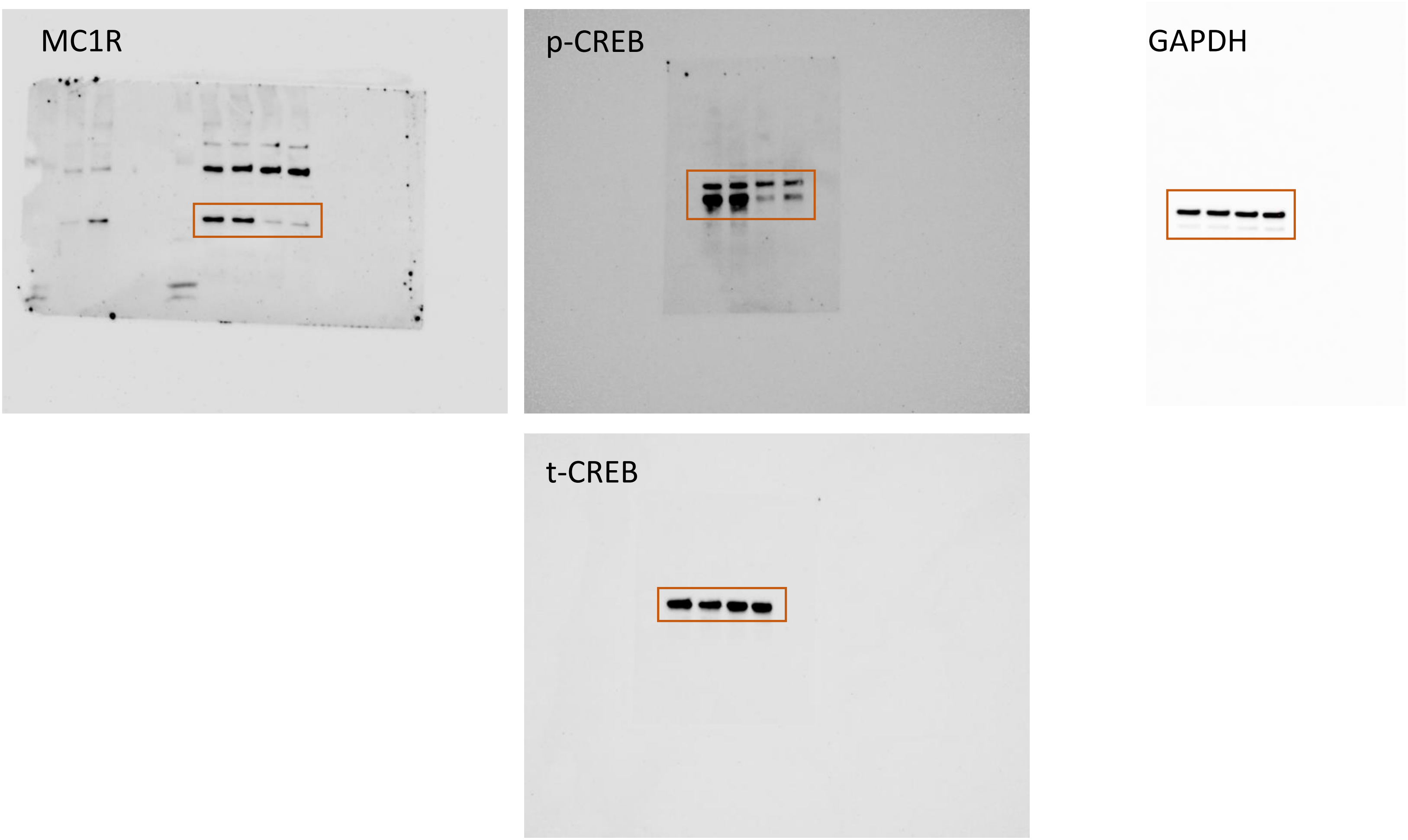

Figure 4c. WT T-47d WT vs. MC1R-KD T-47d cell cycle

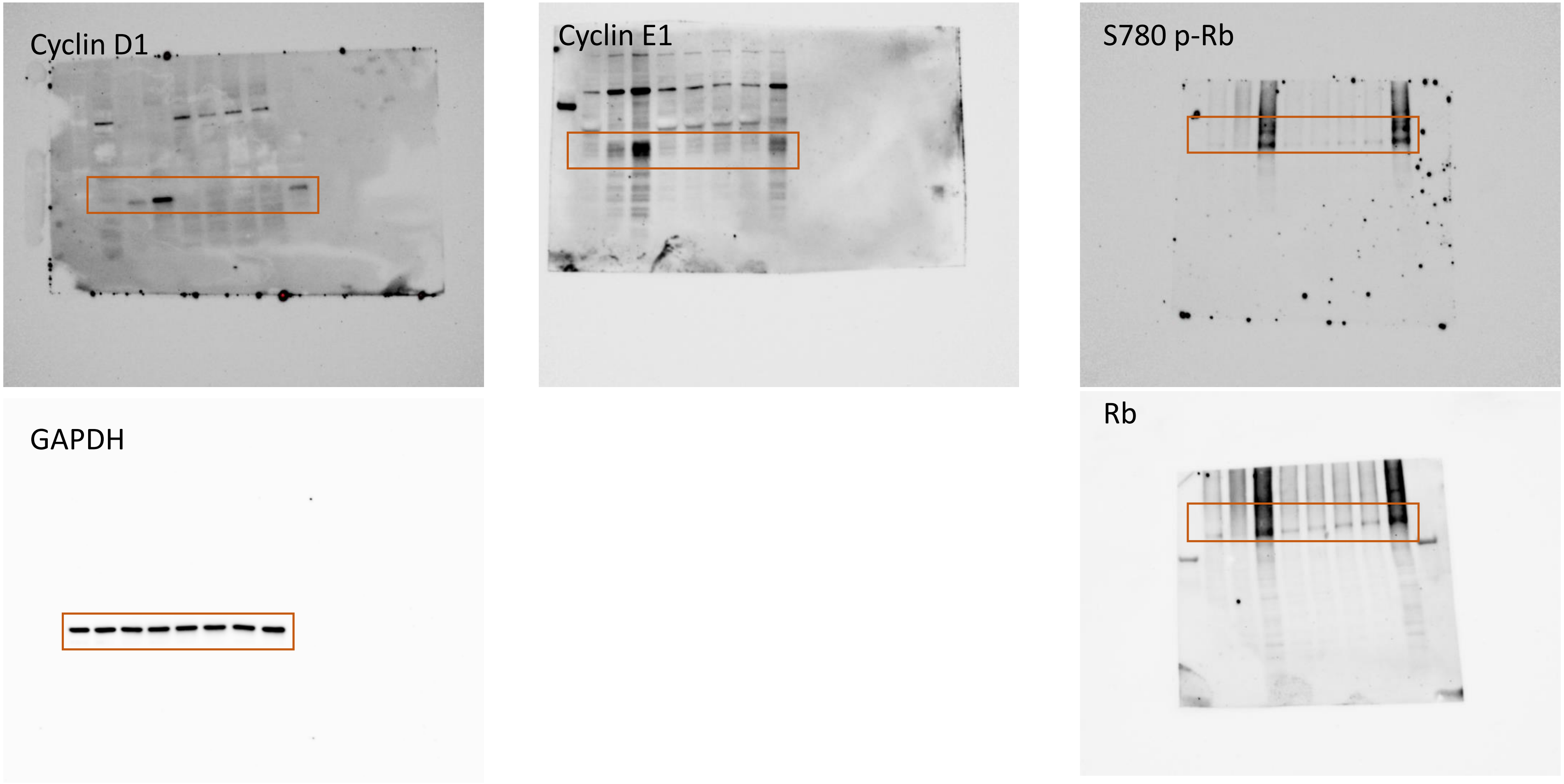

Figure 4f. T47D +/- MSH/MSG cell cycle

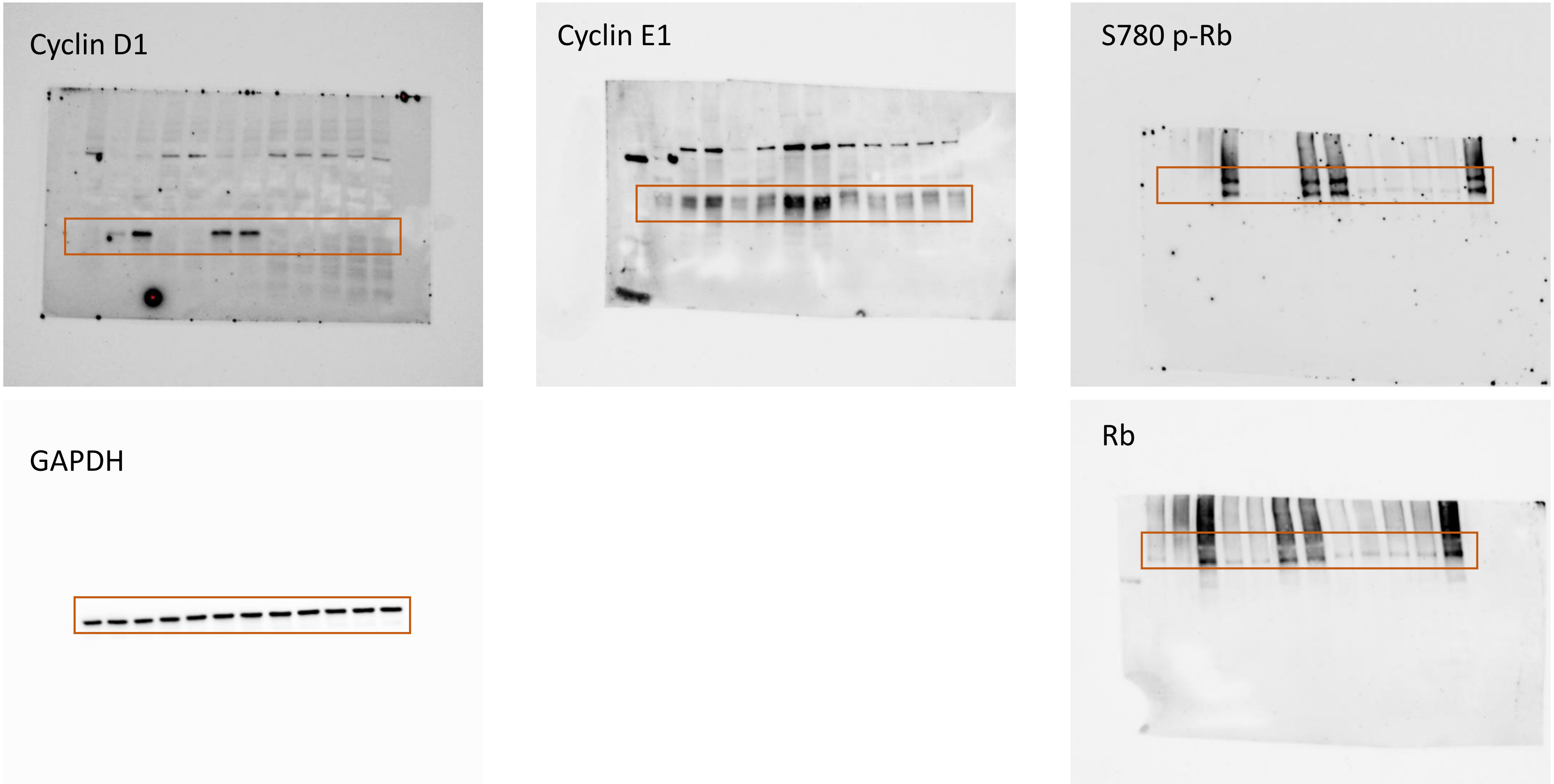

Figure 5c. T47D MC1R KD +/- FSK cell cycle

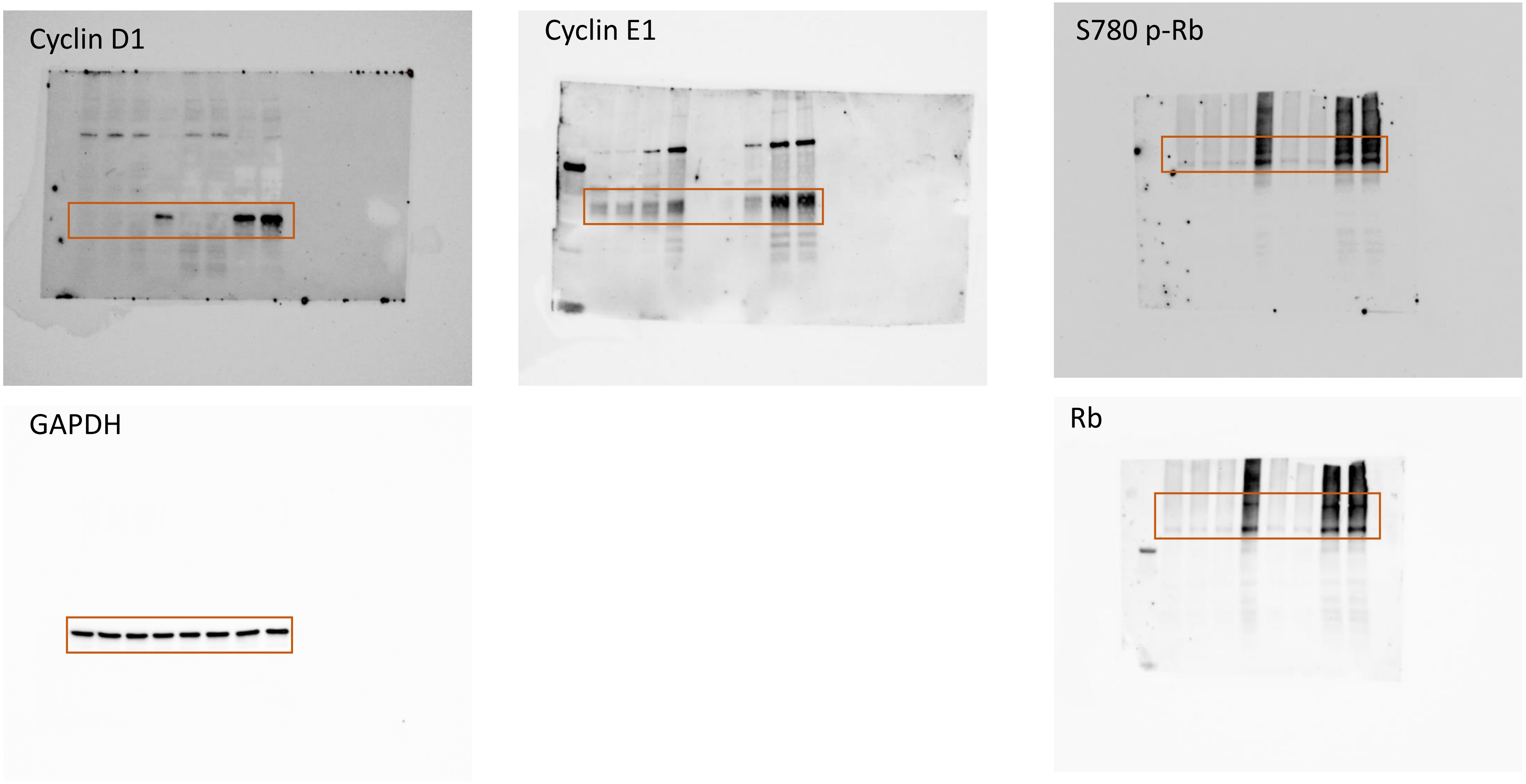

Figure 5f. T47D +/- MSH/CREBi cell cycle

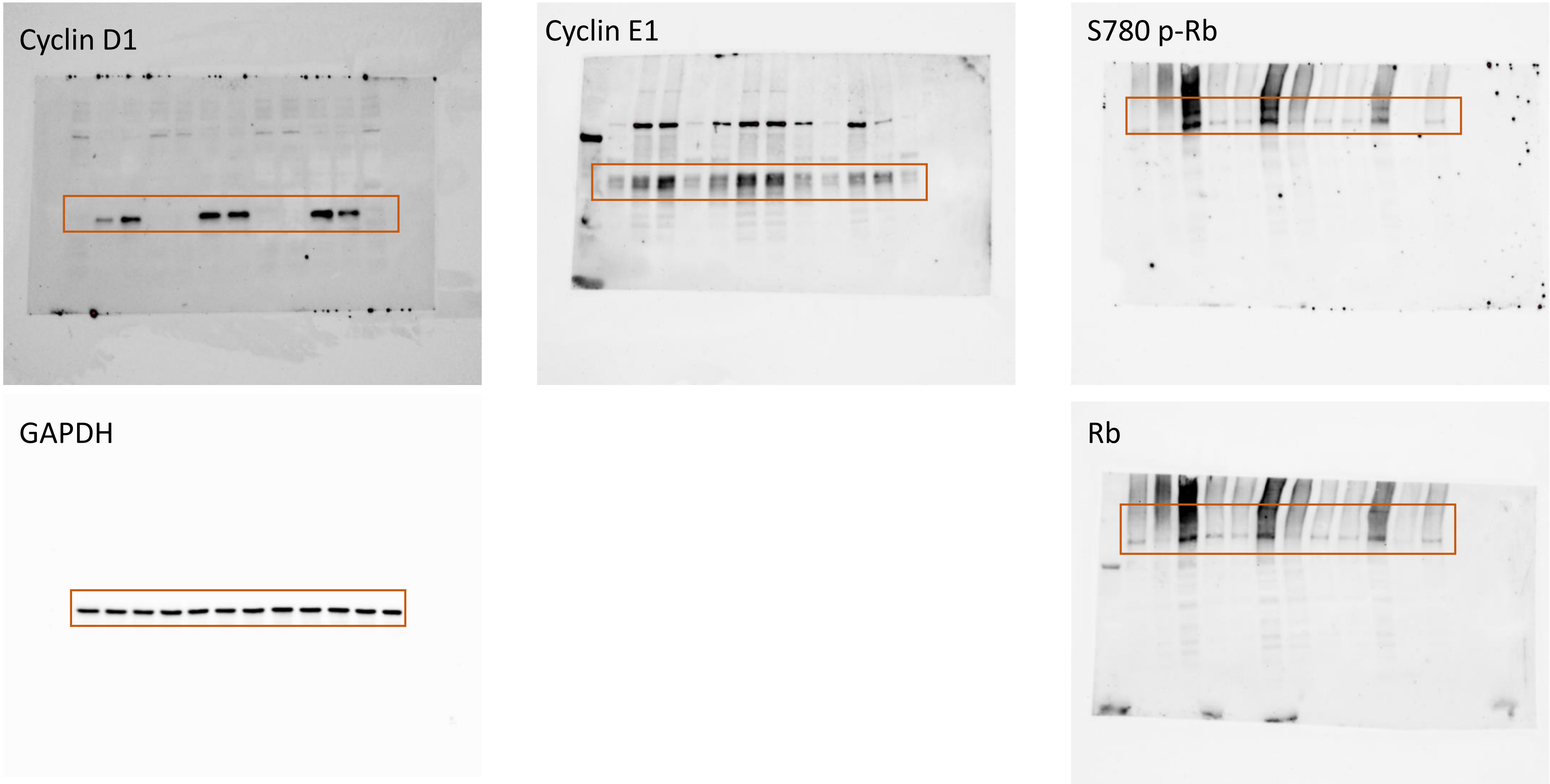

Figure 6a. WT-T-47d and MC1R-KD T-47d downstream signaling

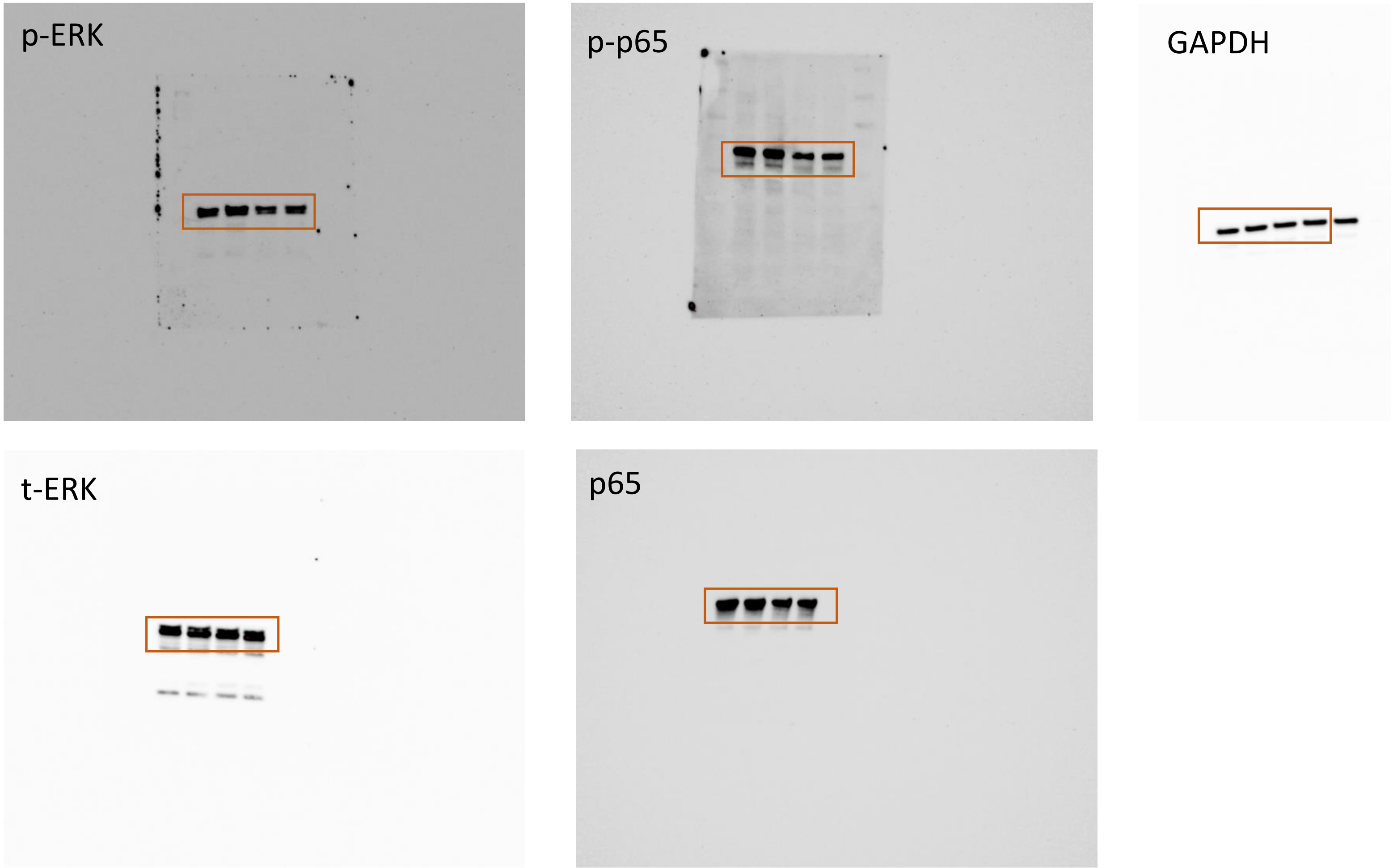

Figure 6b. T47d, MCF7 +/- MSG

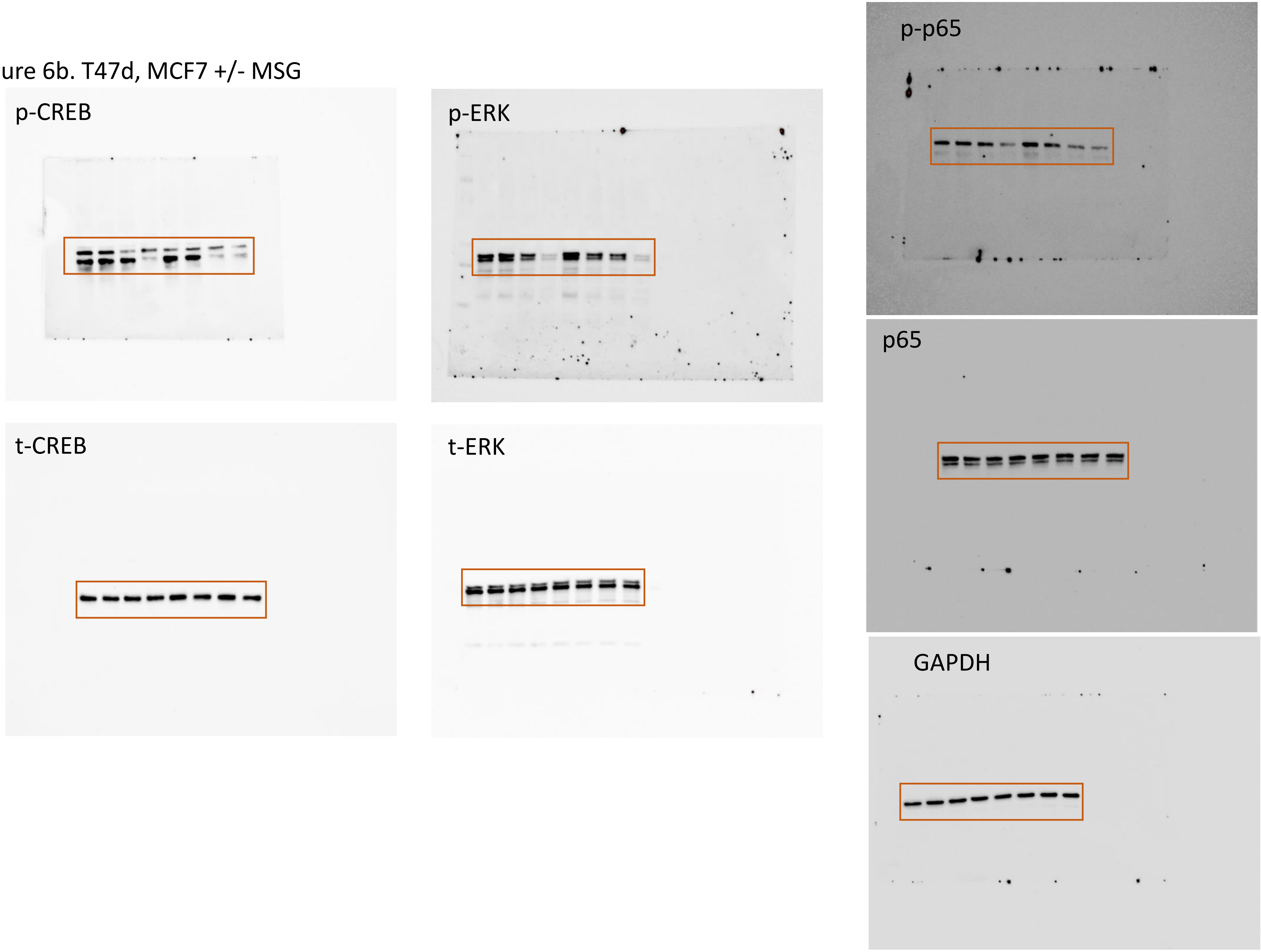

Figure 6c. HEK 293T WT MC1R overexpression

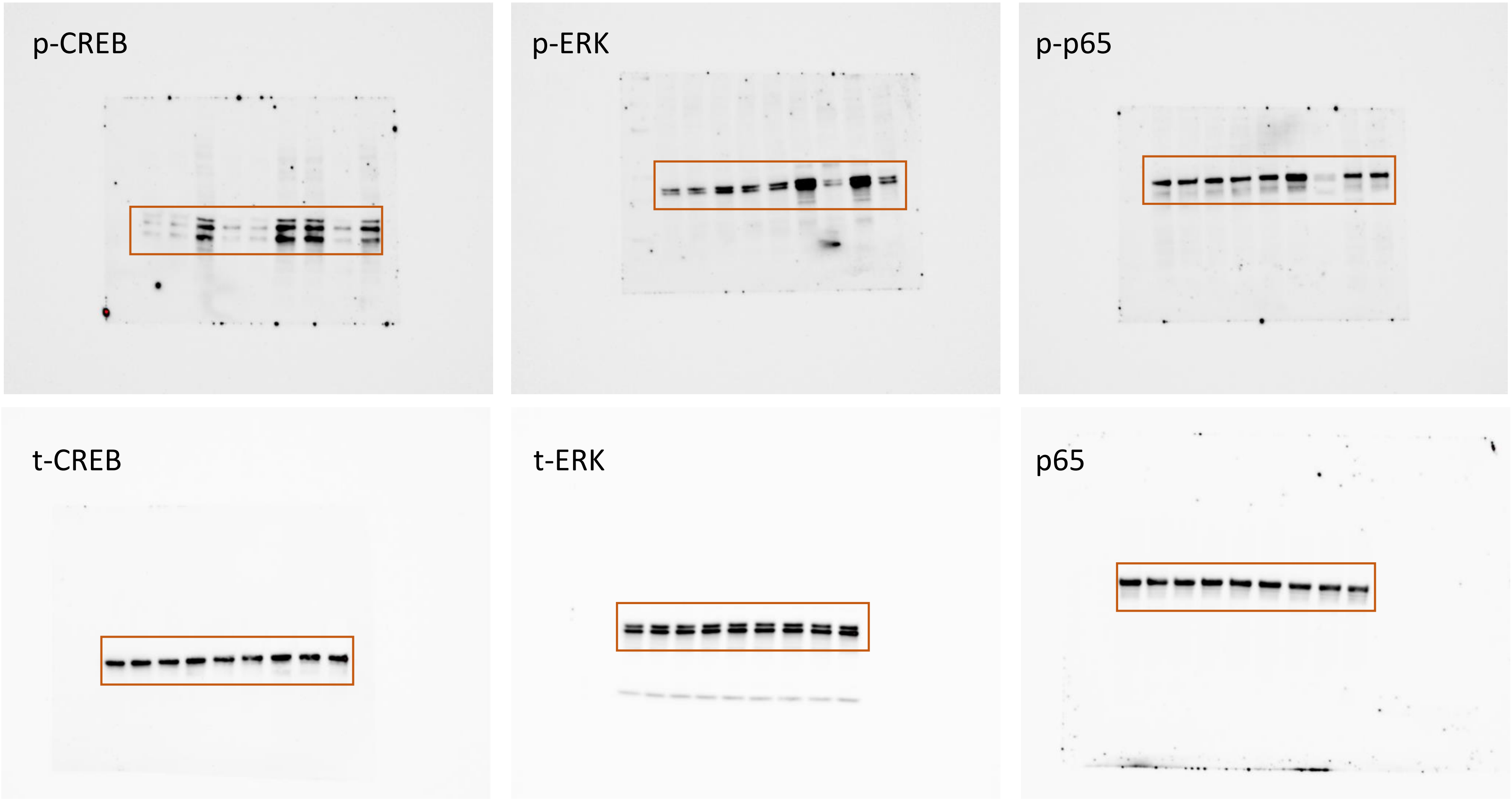

Figure 6c. HEK 293T WT MC1R overexpression. Contd..

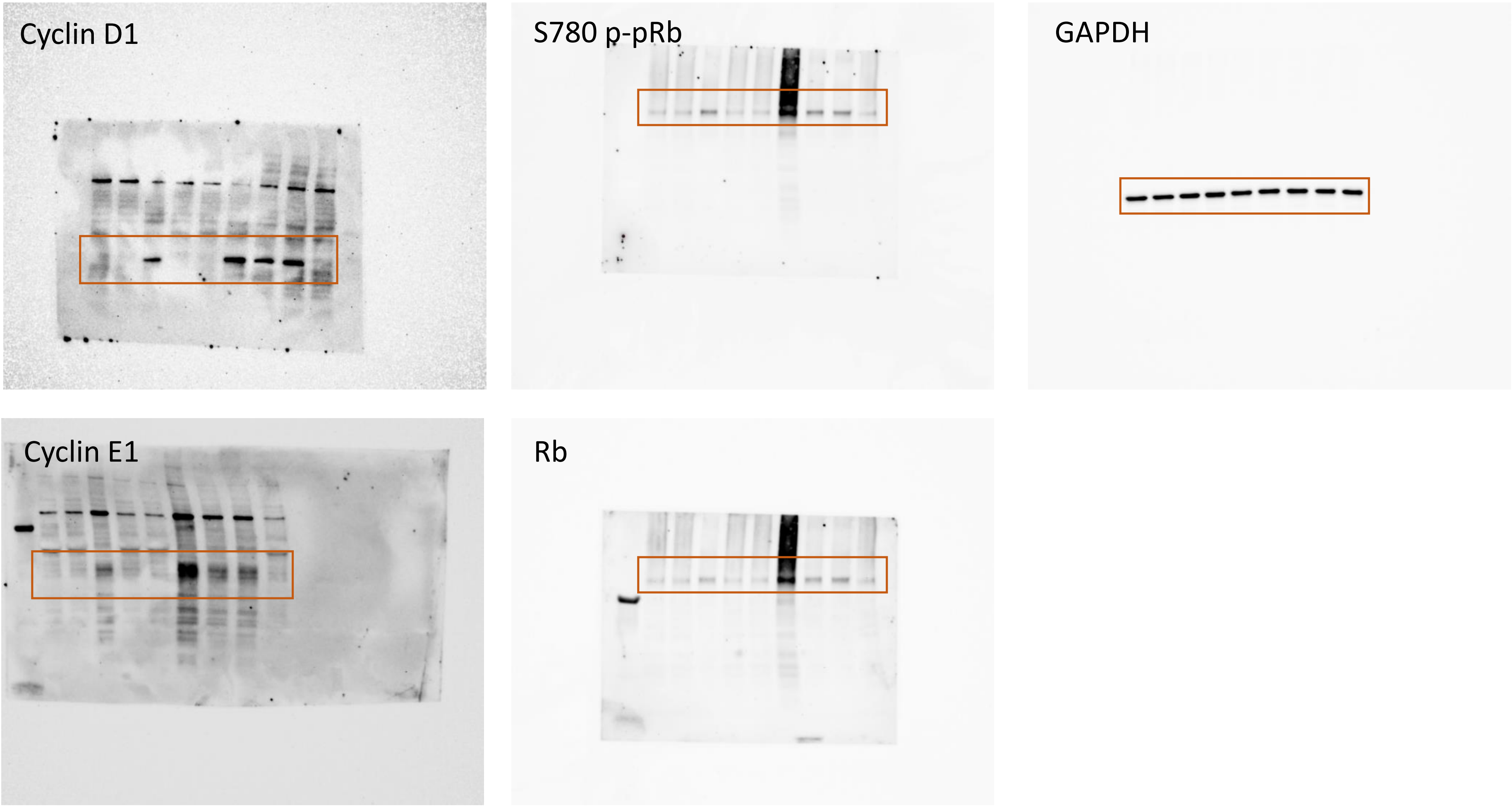

Figure 6d. HEK 293T MC1R WT and variants overexpression

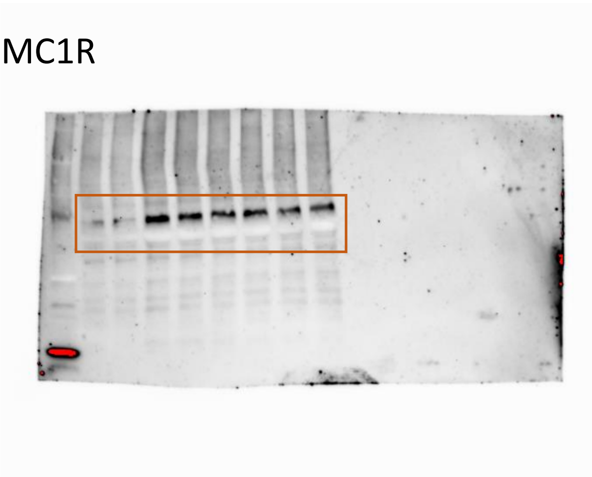

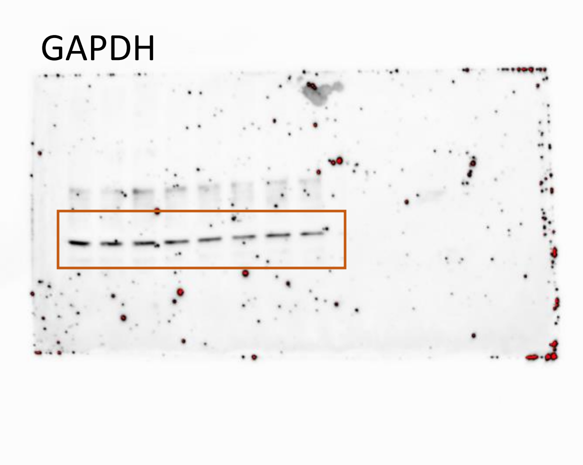

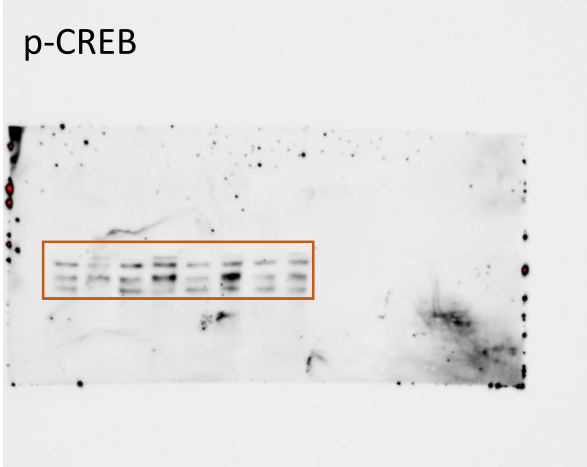

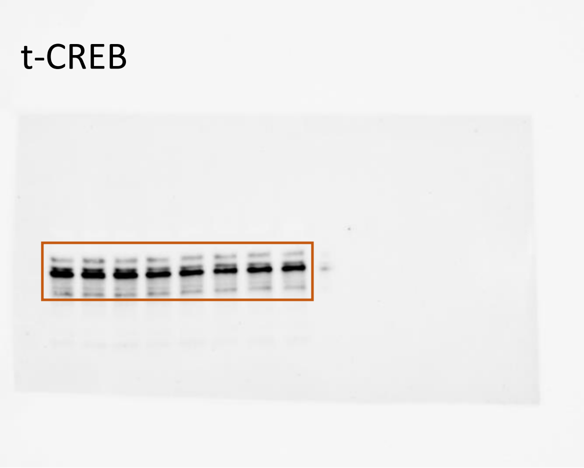

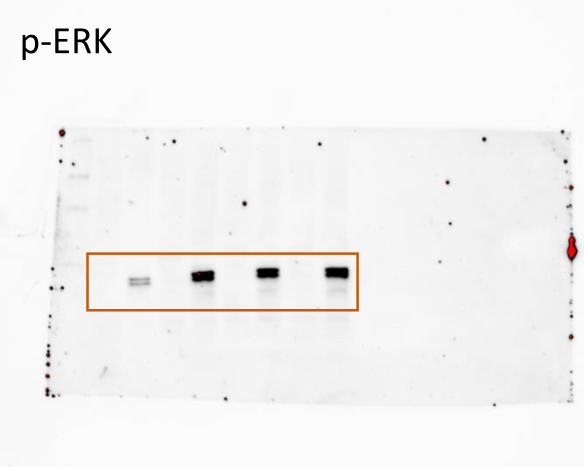

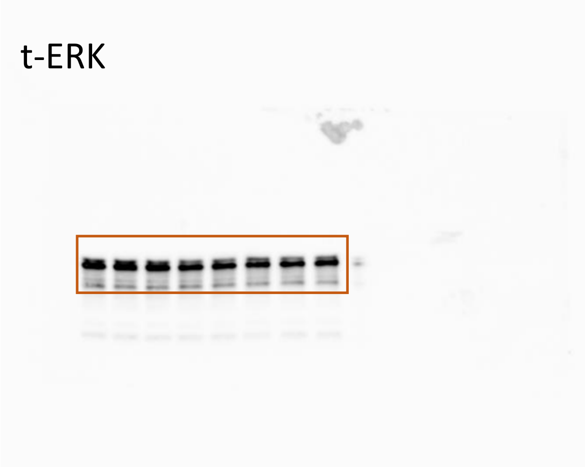

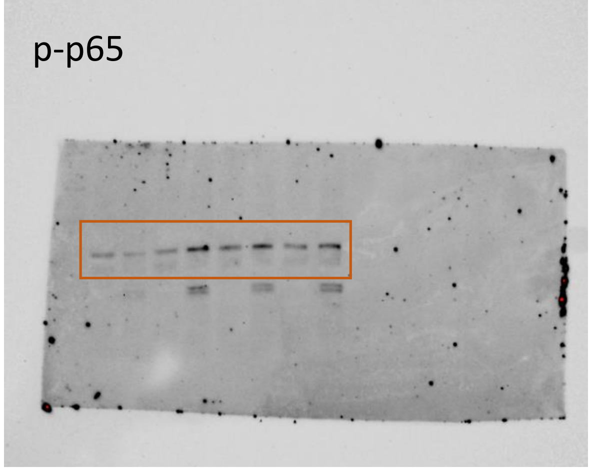

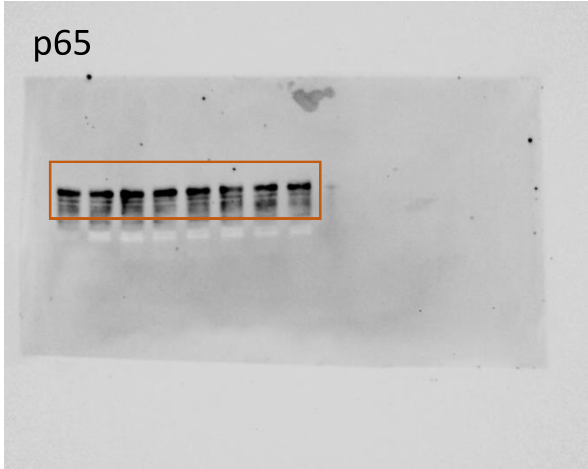

Supplementary Figure 5e. T-47d vs. T-47d+MSH vs. T-47d+MSH+U0126 cell cycle

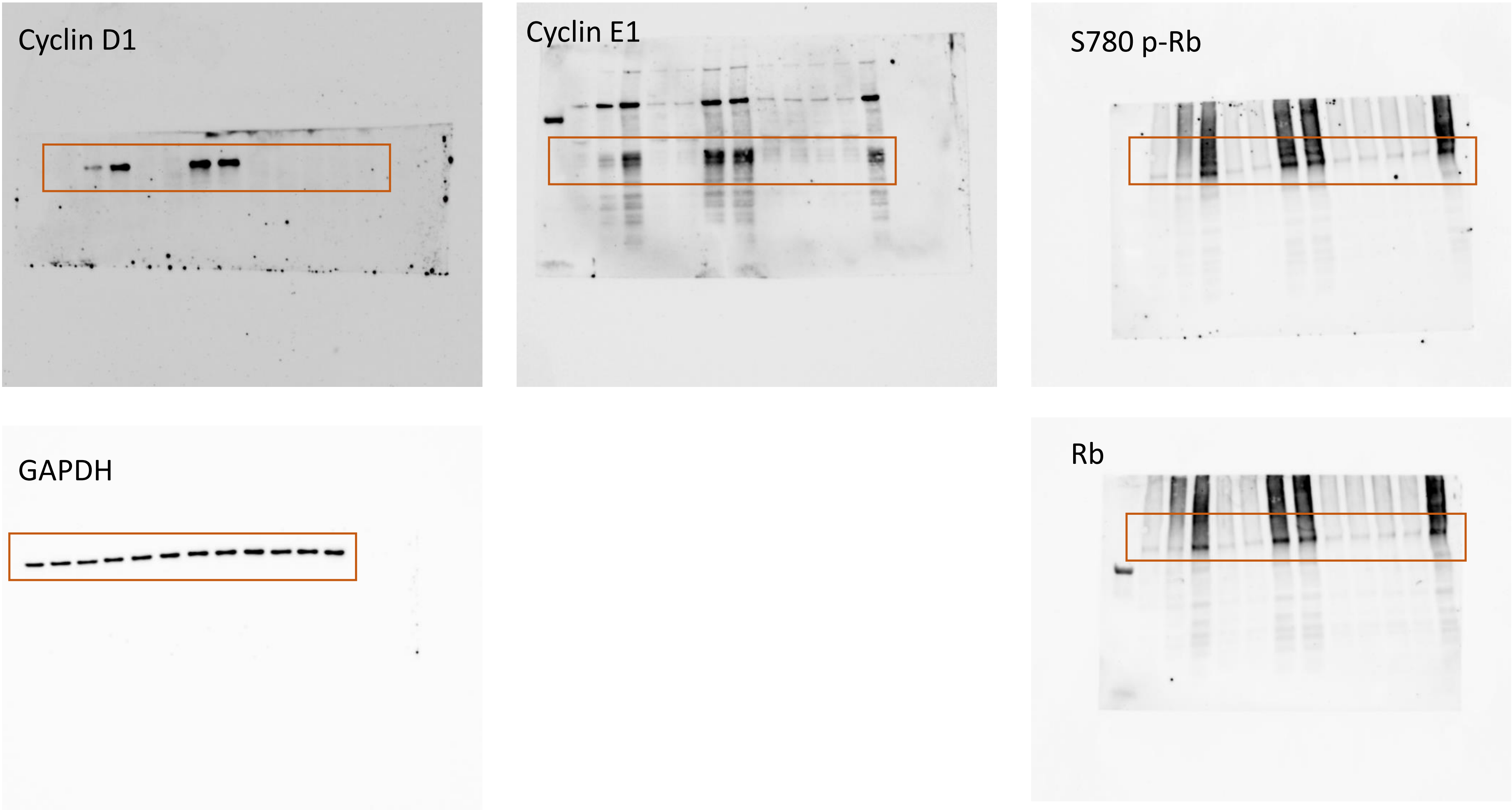

Supplementary Figure 5g. WT T-47d and MC1R-KD-T-47d + FSK

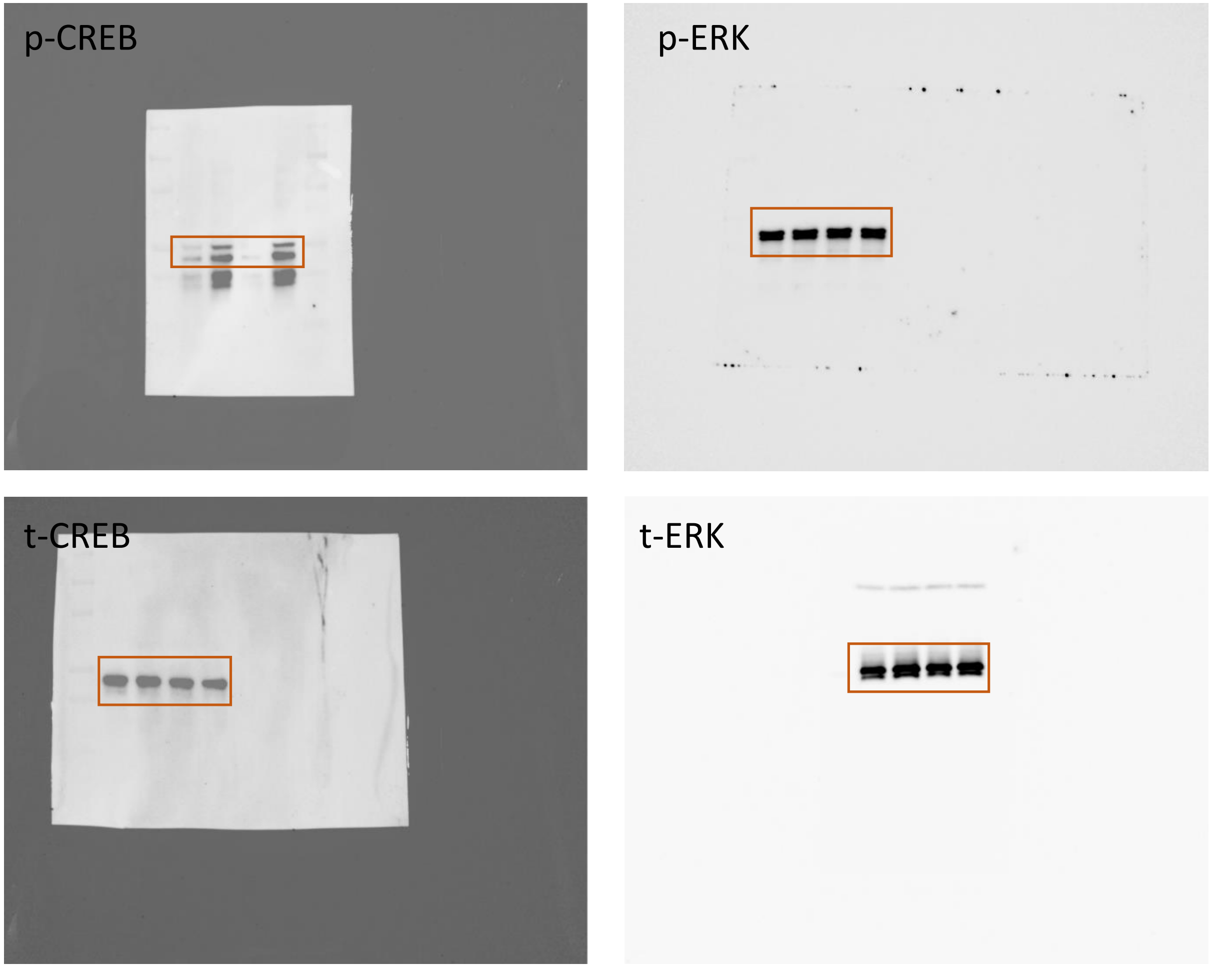

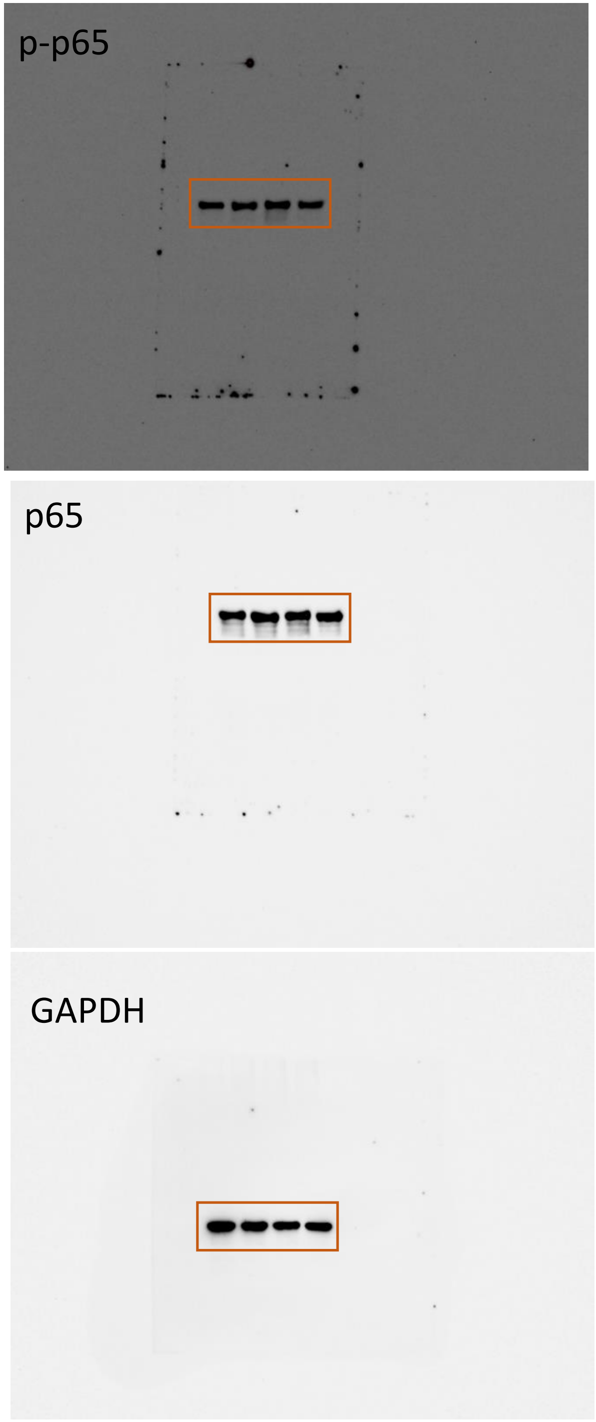

Supplementary Figure 6a. HEK-293T MC1R overexpression

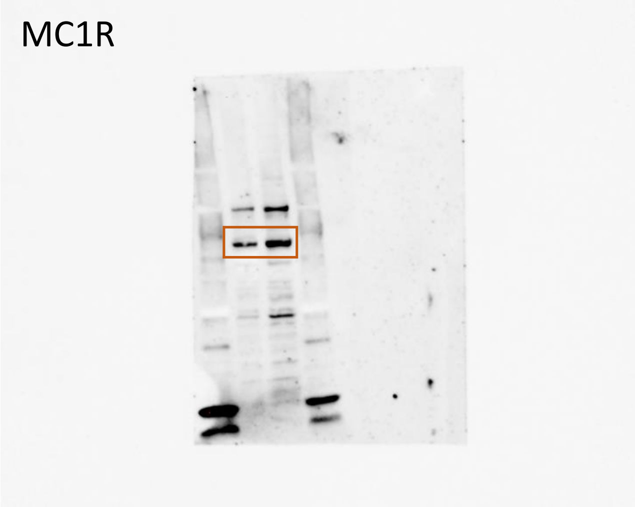

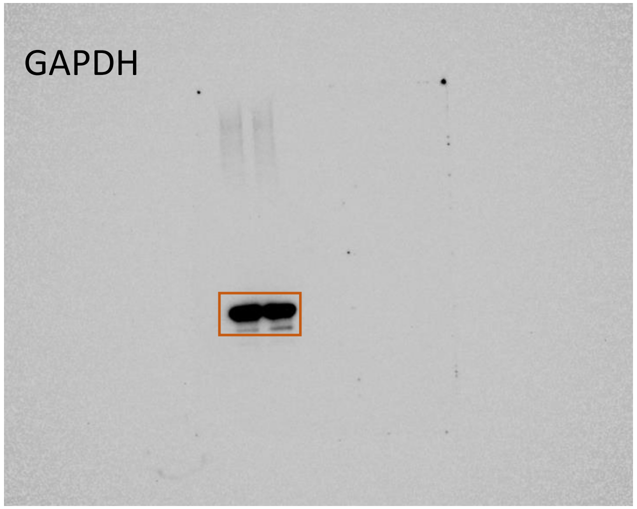

Supplement: Supplementary file 2 — Supplementary Information [file 41698_2023_437_MOESM2_ESM.pdf]
